# Dispersion Stability of Inorganic Powders Harnessed to Mosaic Surface Ligands via Multifit Hansen Solubility Parameters

**DOI:** 10.1021/acs.langmuir.4c00641

**Published:** 2024-07-15

**Authors:** Daisuke Nakamura, Naoko Takahashi

**Affiliations:** Toyota Central R&D Laboratories, Inc., Nagakute, Aichi 480-1192, Japan

## Abstract

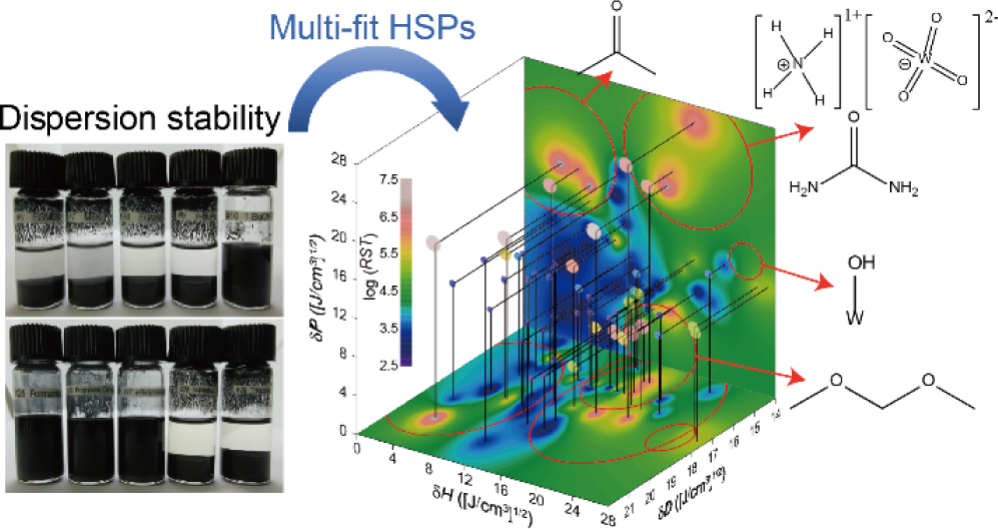

This study assessed the dispersion stability of industrial carbide/oxide powders that have mosaic surfaces comprised of multiple surface ligands. A large number (∼50) of probe liquids were used with an aim to effectively explain the data within the Hansen solubility parameter (HSP) framework. The proposed log-fit method, complemented by multi-HSP analysis featuring harmonic-mean-mixing HSPs, significantly improved the fit to experimental results for various mosaic-surface powders composed of complex surface ligands. X-ray photoelectron spectroscopy and thermal desorption spectroscopy analyses were employed synergistically to decipher the surface ligands of these mosaic-surface powders, which facilitated credible identification and quantification of the surface ligands. These results are in good agreement with the surface ligands and their coverage as determined by the multi-HSP analysis. Consequently, when it comes to characterizing powder surfaces, dispersion stability measurements paired with multi-HSP analysis are superior to conventional XPS and TDS analyses in terms of both topmost surface sensitivity and practicality.

## Introduction

As a surface characterization technique for powders, measurement of the dispersion stability of nano- or submicron-sized particles combined with the concept of Hansen solubility parameters (HSPs) has gained popularity, particularly among the practical formulators of powder-containing products and processes.^1−5^ The dispersion stability of particles in various probe liquids has been determined by measurement of the time taken to reach a certain thickness (approximately a few millimeters) of the supernatant layer during static or centrifuged settling, observed either through visual (naked-eye) observation^6^ or more sophisticated optical instruments such as the LUMisizer.^2,7^ Relaxation-time nuclear magnetic resonance spectroscopy (NMR) analysis, which can directly evaluate the compatibility of a particle–liquid interface, has also been recently introduced as another option for powder measurements.^8^ The dispersion stability of particles in liquids, without the addition of polymeric dispersants, has traditionally been considered to be determined by the Derjaguin–Landau–Verwey–Overbeek (DLVO) mechanism (i.e. electrostatic repulsion among surface-charged particles), mostly in aqueous solutions, or by chemical compatibility (accompanied by very low or negative interfacial energy between particles and liquids), mostly in organic solvents.^3,9,10^ Chemical compatibility between particles and liquids is particularly important for formulators because it provides significant assistance in the determination of which dispersants, solvents, and/or agents should be used for respective applications with reference to the extensive assets of the HSP framework, such as databases, prediction tools, and literature.^6,11^ The HSPs address chemical compatibility between various substances (e.g., organic solvents, polymers,^11,12^ powders,^2^ biomaterials,^13,14^ 2D materials,^15−17^ and ionic liquids^18,19^) through the argument of molecular interaction similarities. These interactions are specifically based on the London dispersion forces (induced dipole–induced dipole interactions), Keesom forces (permanent dipole–permanent dipole interactions), and hydrogen bonding forces, which correspond to δ*D*, δ*P*, and δ*H*, respectively, in the HSP framework. Previous research has highlighted the importance of good similarity/compatibility in HSPs between surface-ligand-controlled particles and the dispersion medium^20,21^ because it significantly enhances the dispersion stability of particles. The HSP framework is thus also expected to be effective in industrial powder characterization. However, industrial powders in the real world are much more complex than laboratory powders with controlled surface ligands. The surfaces of industrial powders can be covered with multiple surface ligands with different chemical compatibilities due to various manufacturing processes (e.g., synthesis, cooling, comminution, classification, washing, and drying). Formulators are unable to modify the surface of industrial powders (for ceramics/cemented-carbide tools) into a controlled single-ligand surface (often due to additional cost); therefore, they often must accept the complexity of industrial powders. Therefore, in this study, we provide combined experimental evidence and a theoretical foundation obtained from the intensive dispersion stability measurements with a significant number of probe liquids to indicate that the HSPs for industrial powders mostly reflect the multiple high-energy surface ligands (nitrogen- and oxygen-containing salts/functional groups) unintentionally generated during the powder manufacturing processes. We consider that these results validate the adoption of the HSP framework for industrial powders, regardless of the complex surfaces with multiple surface ligands.

## Experimental Section

### Materials

Refractory carbides WC and TaC (and their oxides WO_3_ and Ta_2_O_5_ for comparison) were selected as target materials. These materials are commonly used in the production of cemented carbide tools as well as ceramic composites.^22,23^ Two commercial WC powders were used as test powders, a fine WC powder (referred to as WC-AM) with an approximate particle diameter of 1 μm (grade: WC10LV, Allied Material Corporation) and another fine WC powder (referred to as WC-HCS) with an approximate particle diameter of 1 μm (grade: DS100, H. C. Stark Tungsten GmbH). Similarly, two commercial TaC powders were used as test powders, a fine TaC powder (referred to as TaC-HCS) with an approximate particle diameter of 2 μm (H. C. Stark Tungsten GmbH), and another fine TaC powder (referred to as TaC-JNM) with an approximate particle diameter of 2 μm (Japan New Metals Co. Ltd.). Two commercial oxide powders, WO_3_ (referred to as WO_3_-AM) with an approximate particle diameter of 1 μm (grade: F1-WO3, Allied Material Corporation), and Ta_2_O_5_ (referred to as Ta_2_O_5_-KCL) with an approximate particle diameter of 3 μm (grade: TAO02PB, Kojundo Chemical Laboratory Co. Ltd.), were also used as test powders. The scanning electron microscope (SEM) images of the as-received test powders (Figure S1) reveal that these powders consisted not only of primary particles but also of undersized shatters ranging from 50 to 500 nm, which are significantly smaller than the nominal particle diameter (agglomerated or coarse-particle diameter) reported by the suppliers.

### Dispersion and Sedimentation Tests

A small amount of as-received test powders (0.7–3.0 g each to achieve a solid concentration of approximately 5–10 vol % in dispersions) were placed in vials and then immersed and mixed in 2.0 mL of approximately 50 different probe liquids (pure organic solvents covering a wide range of the HSP space). The mixtures were sonicated for 10 min in an ultrasonic cleaning bath to form dispersions (particle size distributions, measured by the laser diffraction method for WC-AM, TaC-HCS, WO_3_-AM, and Ta_2_O_5_–KCL powders in water dispersions, are depicted in Figure S2). The set of probe liquids used for the WC and WO_3_ test powders is shown in Table S1, and that for the TaC and Ta_2_O_5_ test powders in Table S2. The dispersions were left undisturbed for up to 100 days to allow for sedimentation (to reduce sedimentation time significantly, especially for highly viscous probe liquids and nanoparticles, employing a centrifugation method, such as a LUMisizer, would be advantageous). The sedimentation time, *t*_sed_ (in unit of s), for the dispersions (defined as the time taken to reach a clear supernatant layer thickness of 5 mm) was measured through visual observation. The relative sedimentation time (*RST*) was calculated using the following equation:^11^
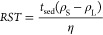
1where ρ_S_ (in unit of g/cm^3^) is the density of solid particles, ρ_L_ (in unit of g/cm^3^) is the density of the probe liquid, and η (in unit of mPa·s) is the viscosity of the probe liquid. The *RST* serves as an indicator of dispersion stability, and is calibrated by the difference in density between the particles and the liquid (which corresponds to the buoyancy force exerted on the particles), as well as the liquid viscosity (which corresponds to the friction force against sedimentation).

### HSP Determination via Sphere and Log-Fit Methods

The HSP distance, *R*_a_, expresses the similarity/compatibility of two different substances (referred to as substance 1 and substance 2) within the HSP framework, and is calculated using the following equation:^11^
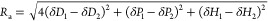
2where the subscripts 1 and 2 refer to HSP components of substance 1 and substance 2, respectively. Smaller *R*_a_ values indicate better similarity/compatibility between the substances, while larger *R*_a_ values indicate poorer similarity/compatibility. The primary method for determining HSPs for particles from the dispersion stability is Hansen’s sphere method. This method involves categorizing the probe liquids into good solvents (those with larger *RST* values than a threshold) or poor solvents (those with smaller *RST* values than a threshold). The threshold, *RST*_th_, used to categorize probe liquids as good/poor solvents, is typically ∼1/50 of the largest *RST* values obtained with respect to the powders as a rule of thumb. In the sphere method, the HSPs for the probe liquids are plotted in 3D-Hansen space (Cartesian coordinates with axes of δ*D*, δ*P*, and δ*H*), and a HSP sphere is obtained by fitting to place the good/poor solvents inside/outside the sphere, of which the center position is the HSP for a powder, and the radius is the interaction radius, *R*_0_.^11^ For single-HSP powders, the single-sphere fitting method is primarily adopted to obtain reliable HSPs for well-controlled powders. In the case of dual-HSP substances, such as gelation agents with both hydrophilic and hydrophobic functional groups,^24−26^ the double-sphere fitting method^6^ is known to effectively determine dual HSPs. This method may facilitate the evaluation of dual-HSP powders.

However, the conventional single/double sphere fitting methods may not be suitable for determining the HSPs for mosaic-surface powders with complex surfaces that consist of 3, 4, or more surface ligands. A new log-fit method is thus proposed to determine the multiple HSPs for mosaic-surface powders. This method utilizes the real-numeric *RST* data obtained from the dispersion and sedimentation tests. A new HSP-distance index for multi-HSP mosaic-surface powders, *R*_a_^Harmonic mean^, is introduced using the harmonic-mean mixing concept, as shown here:
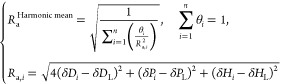
3where *n* is the number of surface ligands, δ*D*_*i*_, δ*P*_*i*_, and δ*H*_*i*_ are the HSP values for the *i*th surface ligand, δ*D*_L_, δ*P*_L_, and δ*H*_L_ are the HSP values for a probe liquid, θ_*i*_ is the surface coverage (or surface-energy share) for the *i*th surface ligand, and *R*_a,*i*_ is the HSP distance from a probe liquid to the respective *i*th surface ligand. This harmonic-mean-mixing concept allows for fitting multiple HSP spheres with variable interaction radii (∝ θ_*i*_). Under the assumption of *n* = 4 or 5, the real-numeric values of log(*RST*) were regressed linearly with respect to *R*_a_^Harmonic mean^ to determine the multiple HSPs (δ*D*_*i*_, δ*P*_*i*_, and δ*H*_*i*_) and their θ_*i*_.

### Other Surface Characterization Techniques

X-ray photoelectron spectroscopy (XPS) and thermal desorption spectroscopy (TDS) were conducted to assist in the identification of surface ligands on the test powders. XPS measurements were performed using an Ulvac-Phi-5500MC spectrometer (X-ray source of Mg–Kα: 1253.6 eV) for the WC powders, and an Ulvac-Phi Versa Probe spectrometer (X-ray source of Al–Kα: 1486.6 eV) for the remaining powders. Wide-scan XPS spectra confirmed that no major impurities were present on the surfaces of any of the test powders, aside from W, Ta, N, O, and C. Narrow-scan X-ray spectra were obtained for the core levels of W 4*f*, N 1*s*, C 1*s*, and O 1*s* for the WC and WO_3_ powders, as well as Ta 4*f*, C 1*s*, O 1*s* for the TaC and Ta_2_O_5_ powders. The spectra were subsequently fitted by curves deconvoluted to potential chemical bonds/surface ligands, which enabled a comparison with the multi-HSP results.

TDS measurements were conducted using an ESCO-TDS1200II spectrometer for the WC-AM (charged sample weight: 9.91 mg), TaC-HCS (3.05 mg), TaC-JNM (3.04 mg), WO_3_-AM (2.94 mg), and Ta_2_O_5_-KCL (2.98 mg) powders to gather supportive data for the surface ligands. The charged sample powders were heated from RT to 600 °C (at calibrated temperatures with a heating rate of 30 °C/min) under ultrahigh vacuum (base background pressure: <10^–7^ Pa). The thermally desorbed molecules were identified using a quadrupole mass spectrometer (*m*/*z*: 1–199). Quantitative analysis was conducted on limited molecules of H_2_, CH_4_, NH_3_, HCN, CO, C_2_H_6_, O_2_, C_3_H_6_, CO_2_, and SO_2_, for which the conversion factors are known.^27^

## Results and Discussion

### HSP Determination via Conventional Single-Sphere Method

**Figure 1:**
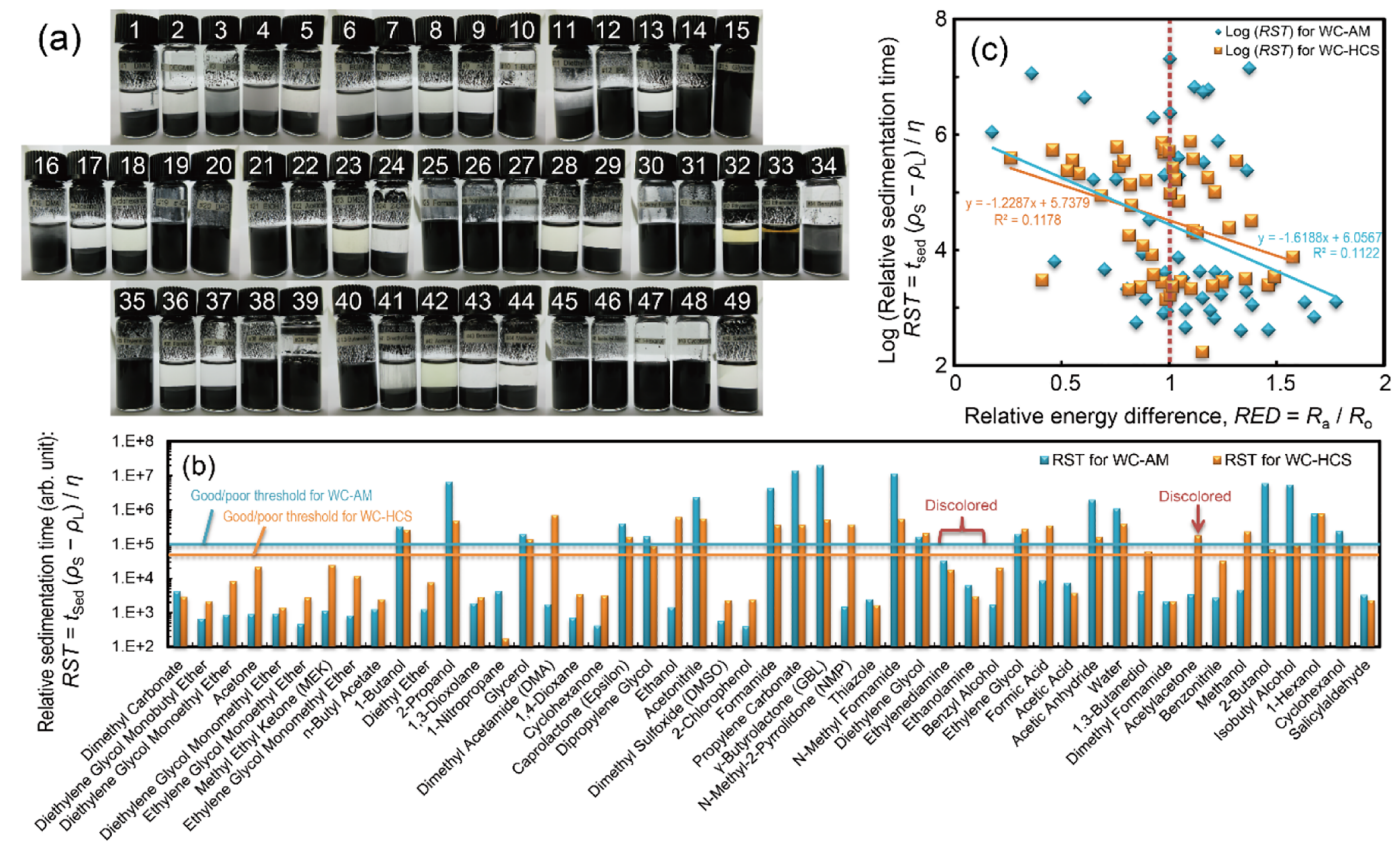
(a) Photographs of dispersions of a WC powder (WC-AM) in all the probe liquids after 10 min sonication and subsequent 1.5 h sedimentation. (b) Relative sedimentation time (*RST*) for WC powders (WC-AM and WC-HCS) in all the probe liquids. (c) Dependence of log(*RST*) for the WC powders in probe liquids on the relative energy difference, *RED* (*R*_a_/*R*_0_), with respect to each HSP obtained for the WC powders (plots for the probe liquids discolored during sedimentation test are excluded).

The WC-AM powder was dispersed in 49 different probe liquids, and subsequent sedimentation after 1.5 h is shown in Figure 1a (see Figure S3 for results of the other powders). The photographs, along with the summarized sedimentation times and *RST* data, detailed in Tables S3–S8 and Figure 1b, effectively show that the dispersions with low- and medium-HSP probe liquids with δ*P <* 10, δ*H* < 10 ([J/cm^3^]^1/2^) flocculated within a few minutes, whereas certain high-HSP probe liquids formed stable dispersions. Additionally, the notably largest *RST* values of ∼10^7^ suggest a Stokes diameter of 100 nm or even less. Thus, the *RST* results align with the smaller diameters of primary particles or undersized shatters rather than those of agglomerated or coarse particles, as illustrated in Figure S2. This highlights a limitation of the sedimentation technique, which tends to focus predominantly on smaller particles present within a diverse size distribution.

This outcome may suggest that only high-HSP surface ligands exist on the industrial carbide powder surfaces. However, both high-HSP surface ligands and a few low- and medium-HSP surface ligands could be present on the carbide powder surfaces, given that most industrial WC and TaC powders have a small quantity of graphitic carbon residues on their surfaces (typically <0.1 wt %, and their HSPs are deemed to be low or medium, resembling other carbon materials with δ*D*: 18.0–19.3, δ*P*: 3.1–6.0, and δ*H*: 3.8–5.3 ([J/cm^3^]^1/2^)),^6^ which originate from the manufacturing processes.^28^ The presence of graphitic carbon surface residues was confirmed by a *sp*^2^-carbon signal in the XPS analysis. This discrepancy, the absence of low-/medium-HSP solvents that can well disperse the powders irrespective of the presence of low- or medium-HSP surface ligands (graphitic carbon surface residues), can be explained via certain solvation effects.^29^

When low- and high-HSP surface ligands coexist on a particle surface, the low-HSP liquids interact poorly with the high-HSP surface ligands. As a result, the high-HSP surface ligands strongly interact with the high-HSP surface ligands on another particle surface, which leads to immediate flocculation. Conversely, high-HSP liquids can strongly interact with high-HSP surface ligands as well as other high-HSP liquids to form a solvation layer near the particle surface. This allows low-HSP surface ligands to be screened out from the low-HSP surface ligands on another particle surface, which leads to a stable dispersion. Thus, only high-HSP liquids can stably disperse the mosaic-surface powders.

**Figure 2:**
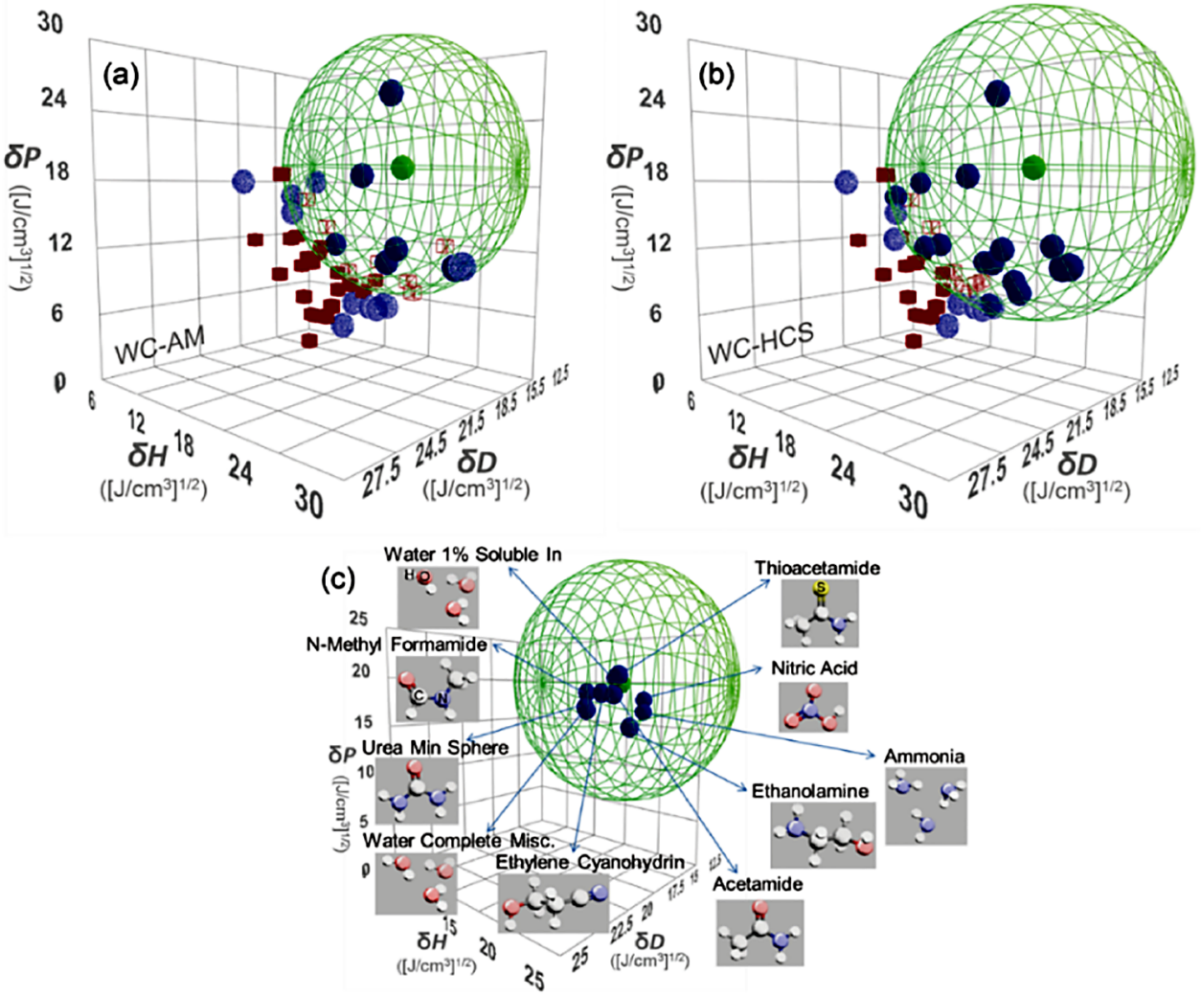
Pseudo-3D plots of good (solid blue circle, open blue circle) and poor (solid red square, open red square) solvents for (a) WC-AM and (b) WC-HCS powders in the HSP space (plots for the dispersions discolored during sedimentation test are excluded). The green wireframe sphere is the outer shell of the HSP sphere fitted via the sphere method, of which the center (green circle) and radius corresponds to the HSP of WC powder and its interaction radius, respectively. The solid and open (or shaded) plots indicate good/poor (normal) and poor/good (anomalous) solvents inside/outside the obtained HSP sphere, respectively. (c) Plots of the HSP sphere for WC-AM powder along with HSPs of the top-10 chemicals (blue circle) closest to the WC-AM powder in the HSP space, of which the molecular structures are represented with ball and stick models.

The *RST* threshold values, *RST*_th_, were established based on the *RST* values in Figure 1b as 100,000 and 50,000 for the WC-AM and WC-HCS dispersions, respectively. This allowed categorization of the probe liquids into good/poor dispersion-stability solvents using the dispersion-stability score (good: 1, poor: 0) outlined in Tables S3 and S4 (the scores for the other powders are listed in Tables S5–S8). The single-sphere method was applied to the good/poor dispersion-stability scores to determine the HSPs for the test powders. Figure 2a,b show the sphere fitting results for the WC-AM and WC-HCS powders (the results for the TaC and oxide powders are shown in Figures S4 and S5).

Although the derived HSP spheres in Figure 2a,b were established to place the good/poor solvents inside/outside the sphere, respectively, there were a considerable number of anomalous values (good/poor solvents outside/inside the sphere, respectively). The obtained HSP, *R*_0_, and FIT^11^ values are summarized in Table 1. The FIT values for WC-AM, WC-HCS, and TaC-HCS were significantly below the perfect-fit result of FIT = 1, due to the presence of a large number of anomalous values. This suggests that multiple surface ligands caused multiple HSPs, which led to the poor fitting quality.

**Table 1:** Summary of HSPs for Test Powders Determined via the Conventional Single-Sphere Method, their Possible Surface Ligands, and FIT values (FIT = 1 Indicates Perfect Fitting Quality Without Anomalous Solvents)

test powder, material-supplier	δ ([J/cm^3^]^1/2^)	δ*D* ([J/cm^3^]^1/2^)	δ*P* ([J/cm^3^]^1/2^)	δ*H* ([J/cm^3^]^1/2^)	*R*_0_ ([J/cm^3^]^1/2^)	possible surface ligand	FIT	number of good solvents / total probe liquids
WC-AM	31.0	15.6	19.6	18.2	12.2	Ammonium salt	0.252	17/46 (*RST*_th_ = 100,000)
WC-HCS	32.0	14.9	19.7	20.3	14.6	Ammonium salt	0.386	23/45 (*RST*_th_ = 50,000)
TaC-HCS	34.7	20.2	14.8	24.0	13.8	Urea and/or hydroxide	0.392	14/47 (*RST*_th_ = 50,000)
TaC-JNM	31.7	19.0	23.1	10.4	9.8	Ester, amide, and/or ketone	0.952	6/42 (*RST*_th_ = 400,000)
WO_3_-AM	30.7	17.3	22.2	12.4	11.1	Ester, amide, and/or ketone	0.935	9/46 (*RST*_th_ = 200,000)
Ta_2_O_5_-KCL	25.2	19.4	14.0	8.1	5.8	Ester, amide, and/or ketone	0.876	7/48 (*RST*_th_ = 16,000)

To identify potential surface ligands on the WC-AM powder, the HSP database was referenced to match the single-sphere HSP values of δ*D*: 15.6, δ*P*: 19.6, and δ*H*: 18.2 ([J/cm^3^]^1/2^). The top-10 chemicals closest to them were determined with HSP sets of low δ*D*, high δ*P*, and high δ*H* (such as water, ammonia, amide, and amine), as shown in Figure 2c. The top-2 chemicals are water (from 1% soluble-in data) and ammonia, which are constituents of ammonium salts (e.g., ammonium tungstate). This suggests the presence of an ammonium salt as a dominant surface ligand on the WC-AM (as well as WC-HCS) powder surface, in accordance with the popular like-seeks-like^11^ concept in the HSP framework (here the concept is extended to like-solvates-like).

Table 2 lists the reference chemicals and their HSPs used to assist the identification of potential surface ligands.^6^ According to this, the dominant surface ligands were tentatively identified as urea and/or hydroxide for the TaC-HCS powder, and ester, nitrile, amide, and/or ketone for the TaC-JNM, WO_3_-AM, and Ta_2_O_5_-KCL powders. While the single-sphere method is still useful to form a broad picture of powder surface characteristics, there is much room for improvement. Figure 1c clearly shows the poor correlation (with a determination coefficient of *R*^2^ ∼ 0.1) of the *RST* values for the WC powders to the relative energy difference (*RED* = *R*_a_/*R*_0_) values. This suggests that real-numeric *RST* values and multiple HSPs for mosaic-surface powders should be considered for HSP determination to ensure a better understanding of powder surfaces.

**Table 2:** List of Reference Chemicals and their HSPs to Identify the Possible Surface Ligands for HSPs Determined via the Conventional Single-Sphere Method as well as the Log-Fit Method with Multiple HSPs

reference to	reference chemical from HSP database	δ ([J/cm^3^]^1/2^)	δ*D* ([J/cm^3^]^1/2^)	δ*P* ([J/cm^3^]^1/2^)	δ*H* ([J/cm^3^]^1/2^)	Remarks
Ammonium salt	Ammonia	28.6	13.7	16.7	18.8	Low δ*D*, high δ*P*, high δ*H*
	Water 1% soluble in—Ro = 18.1	30.3	15.1	20.4	16.5	
Urea	Urea (min sphere)	29.4	17.6	17.3	16.0	Medium δ*D*, high δ*P*, high δ*H*
Amine	Ethylenediamine	25.3	16.6	8.8	17.0	Medium δ*D*, medium δ*P,* high δ*H*
Hydroxide	Methanol	29.4	14.7	12.3	22.3	Medium δ*D*, medium δ*P*, high δ*H*
	Ethylene Glycol	33.0	17.0	11.0	26.0	
Carbonate salt	Mg[NO_3_]_2_·6H_2_O	36.7	19.5	22.1	21.9	High δ*D*, high δ*P*, high δ*H*
Ether and/or alcohol	Propylene glycol monobutyl ether	18.4	15.3	4.5	9.2	Medium δ*D*, low δ*P*, medium δ*H*
	Methylal (dimethoxy methane)	17.4	15.0	1.8	8.6	
Ester	Ethylene carbonate	28.7	18.0	21.7	5.1	Medium δ*D*, high δ*P*, low δ*H*
Amide	Dimethylformamide (DMF)	24.9	17.4	13.7	11.3	Medium δ*D*, high δ*P*, medium δ*H*
Nitrile	Acetonitrile	24.4	15.3	18.0	6.1	Medium δ*D*, high δ*P*, low δ*H*
Ketone	Acetone	19.9	15.5	10.4	7.0	Medium δ*D*, high δ*P*, low δ*H*

### HSP Determination via Log-Fit Method with Multiple HSPs

The real-numeric log(*RST*) values were used to determine multiple HSPs for mosaic-surface powders with the assistance of eq 3. The harmonic-mean-mixing distance, *R*_a_^Harmonic mean^, was correlated with the log(*RST*) values. A linear regression using the least-squares method was subsequently conducted to achieve the best correlation between them with variable multi-HSP values and their coverages (δ*D*_*i*_, δ*P*_*i*_, δ*H*_*i*_, θ_*i*_ for *i* = 1···, *n*, where *n* = 4 or 5) as fitting parameters. Once the optimal correlation was attained (as depicted in Figure S6, which now includes an updated determination coefficient *R*^2^ of ∼0.7, reflect a considerable improvement upon single-sphere fit, which only managed an *R*^2^ of ∼0.1), the variable fitting parameters were deemed as the true multi-HSP values for potential surface ligands and their coverages. Table 3 summarizes the HSPs and their coverages for surface ligands for the test powders derived via the real-numeric log(*RST*)-fit method with multiple HSPs.

**Table 3:** Summary of HSPs for Test Powders Determined via theLog-Fit Method with Multiple HSPs, Coverages, and Potential Surface Ligands

test powder, material-supplier	possible surface ligand	coverage, θ (%)	δ ([J/cm^3^]^1/2^)	δ*D* ([J/cm^3^]^1/2^)	δ*P* ([J/cm^3^]^1/2^)	δ*H* ([J/cm^3^]^1/2^)
WC-AM	Ammonium salt + urea + amine	36.2	33.5	16.9	22.4	18.3
	Ketone + amide	32.7	26.6	18.0	19.3	3.8
	Ether and/or alcohol	20.5	21.6	15.2	2.3	15.2
	Hydroxide	10.6	33.2	17.6	13.4	24.7
	Anhydride	0.001	22.3	16.0	11.7	10.2
WC-HCS	Hydroxide + ammonium salt + urea + amine	53.8	35.7	15.9	19.8	25.0
	Ketone + amide	29.4	26.7	17.3	20.3	0.0
	Ether and/or alcohol	16.1	20.8	14.8	0.0	14.6
	Amide	0.7	22.5	17.3	11.6	8.6
TaC-HCS	Ether and/or alcohol	32.9	22.4	15.8	0.0	16.0
	Hydroxide	27.0	30.2	15.6	10.7	23.5
	Ketone + amide	24.0	25.5	18.8	16.7	4.1
	Urea + amine	16.1	30.8	16.4	20.2	16.5
TaC-JNM	Carbonate salt	58.4	41.0	19.0	25.0	26.4
	Ester + ketone	33.9	27.1	18.9	19.0	3.8
	Ether and/or alcohol	7.7	22.5	17.2	0.0	14.6
	Amide	0.01	30.2	17.4	18.8	15.9
WO_3_-AM	Nitrile	64.1	26.8	18.5	18.8	4.2
	Hydroxide + urea + amine	24.7	31.8	16.4	12.9	24.0
	Ether and/or alcohol	11.2	22.7	17.1	1.8	14.8
	Amide	0.004	30.2	17.4	18.8	15.9
	Anhydride	0.002	22.3	16.0	11.7	10.2
Ta_2_O_5_-KCL	Urea + amine	41.8	29.3	14.3	21.6	13.7
	Nitrile	40.0	26.1	18.9	15.6	9.0
	Ether and/or alcohol	10.6	23.4	18.6	2.8	13.9
	Hydroxide	7.5	31.9	17.3	9.5	25.1

**Figure 3:**
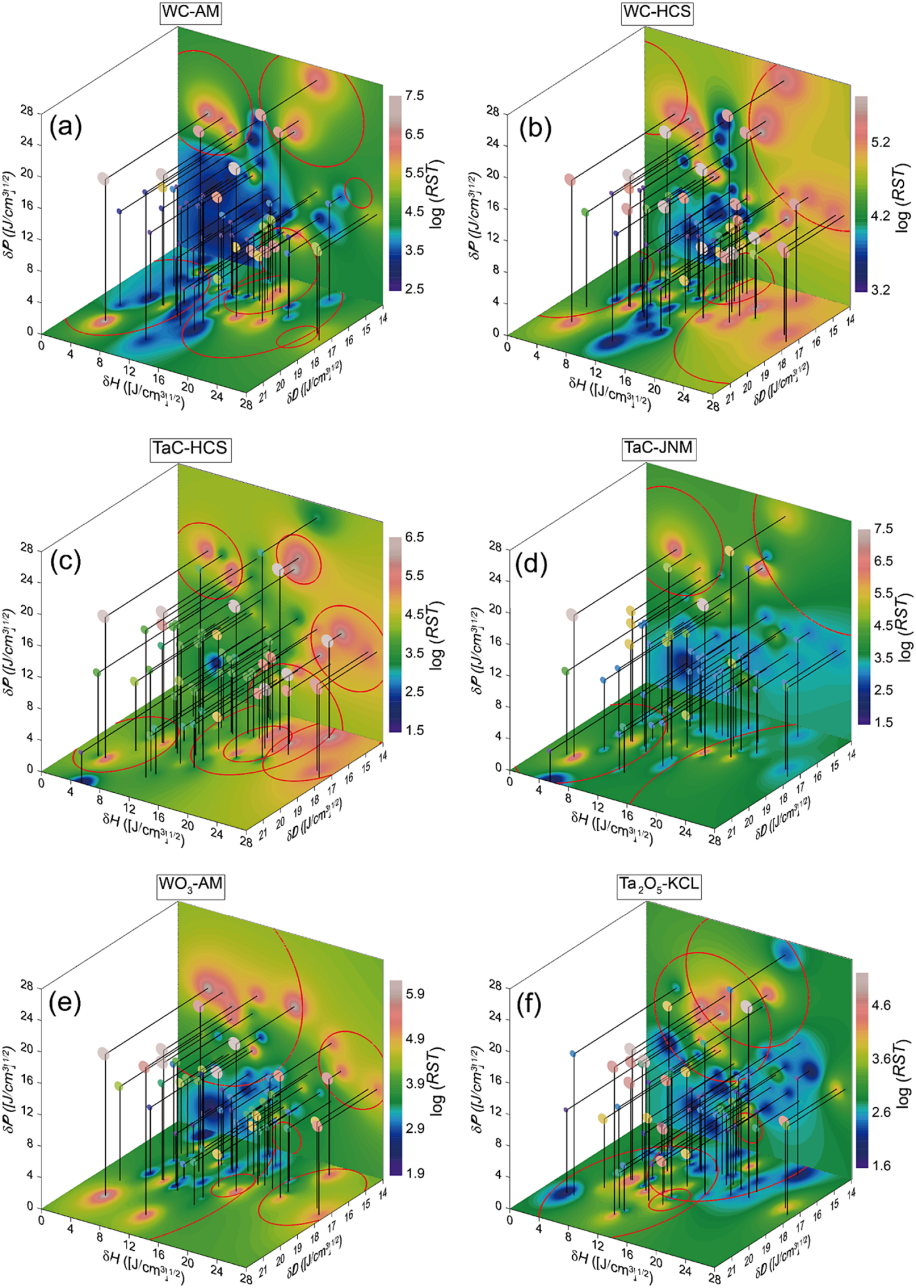
Pseudo-4D plots of probe liquids in HSP space, where *RST* values for powders of (a) WC-AM, (b) WC-HCS, (c) TaC-HCS, (d) TaC-JNM, (e) WO_3_-AM, and (f) Ta_2_O_5_-KCL are color-coded (good dispersion stability: gray–red, intermediate dispersion stability: yellow–green, poor dispersion stability: blue–purple). The projection of the *RST* values to the δ*H*–δ*D* and δ*H*–δ*P* planes is drawn in the same color as that in the pseudo-4D plots; the data-deficit region was interpolated using a graphing software. The HSPs (and coverages) for surface ligands on topmost powder surfaces were determined via linear regression using the harmonic-mean-mixing method, pseudo interaction radii *R*_0_, which were set to 20 times the coverage *θ*_*i*_. The red circles represent the projection of the HSP spheres for the surface ligands with the pseudo *R*_0_ to the *δH*–*δD* and *δH*–*δP* planes.

Figure 3 presents pseudo-4D plots of the HSPs and log(*RST*) values for the probe liquids and pseudo HSP spheres (rendered in red circles) with pseudo interaction radii *R*_0_, set to 20 times the coverage θ_*i*_. This effectively replicates the log(*RST*), i.e. the distribution of good/poor dispersion-stability solvents. As a result, the log-fit method combined with the harmonic-mean-mixing distance and multiple HSPs was confirmed as being highly effective for dealing with large volumes of real-numeric *RST* data for dispersions.

Comparing the derived multiple HSPs with those for the reference chemicals listed in Table 2 facilitated the identification of potential surface ligands, as shown in Table 3 (the following XPS and TDS results were also used for identification purposes). Some of the ligand identifications were almost identical to those obtained by the single-sphere method (for example, ammonium salt for the WC-AM powder), whereas others varied significantly (for instance, carbonate salt for TaC-JNM by the log-fit method with multiple HSPs versus ester, nitrile, amide, and/or ketone by the single sphere method). The validity of the surface-ligand identification and the coverage quantification was verified through the following consideration of the XPS and TDS results.

### XPS Analysis

**Figure 4:**
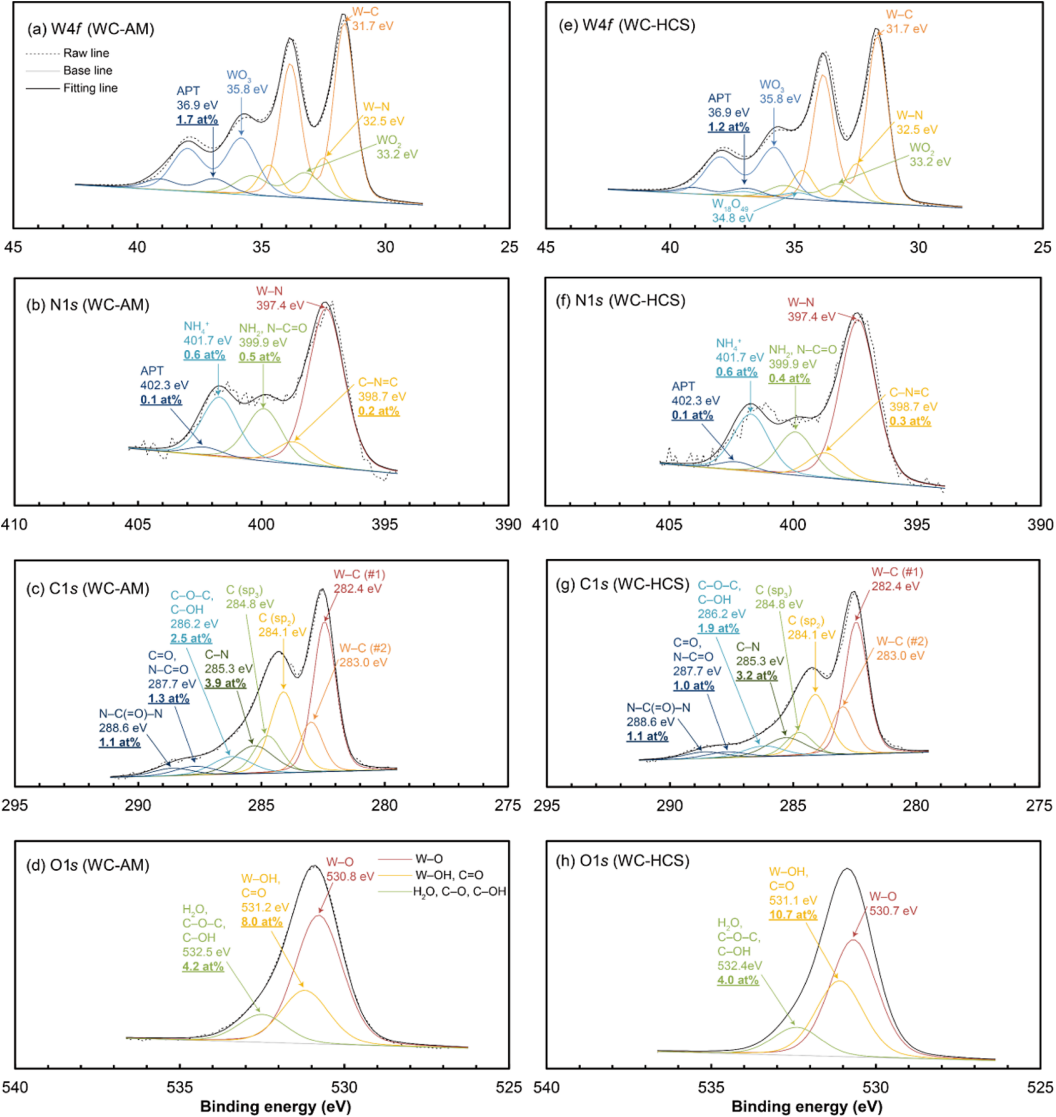
XPS spectra obtained from the (a–d) WC-AM and (e–h) WC-HCS powders and their deconvoluted and convoluted fitting curves for core levels of (a,e) W 4*f*, (b,f) N 1*s*, (c,g) C 1*s*, and (d,h) O 1*s*.

**Figure 5:**
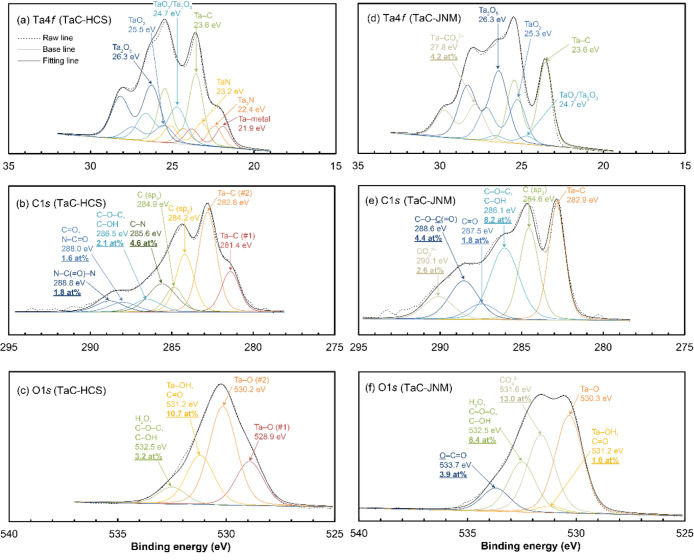
XPS spectra obtained from the (a–c) TaC-HCS and (d–f) TaC-JNM powders and their deconvoluted and convoluted fitting curves for core levels of (a,d) Ta 4*f*, (b,e) C 1*s*, and (c,f) O 1*s*. N 1*s* spectra were omitted due to their peak superposition with strong Ta 4*p* peaks.

**Figure 6:**
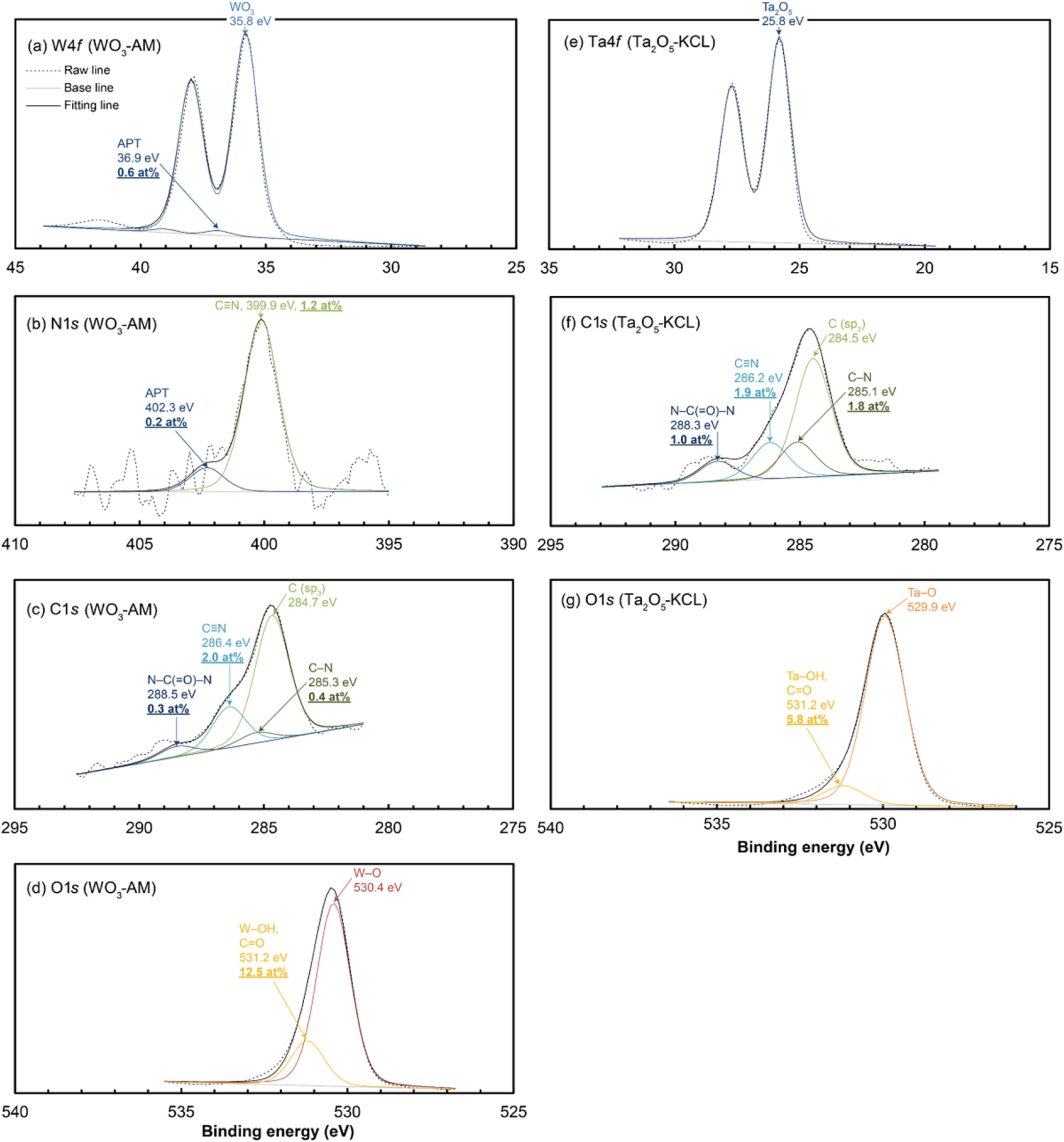
XPS spectra obtained from the (a–d) WO_3_-AM and (e–g) Ta_2_O_5_-KCL powders and their deconvoluted and convoluted fitting curves for core levels of (a) W 4*f*, (b) N 1*s*, (c,f) C 1*s*, (d,g) O 1*s*, and (e) Ta 4*f*. The N 1*s* spectrum for Ta_2_O_5_-KCL was omitted due to its peak superposition with the strong Ta 4*p*_2/3_ peak.

Narrow-scan XPS spectra obtained from the WC, TaC, and oxide powders are shown in Figures 4–6, respectively. The spectra were deconvoluted to identify and quantify chemical bonds and surface ligands, with the results summarized in Table S9.

### WC Powders

The XPS spectra for WC-AM and WC-HCS were essentially the same with only slight differences in surface ligand concentration. This aligns with their similar multiple HSPs as listed in Table 3, which is why the TDS measurement for the WC-HCS powder was omitted.

Figure 4a,e illustrate the chemical shifts in the W 4*f*_7/2_ peaks. Not only do these spectra suggest the presence of carbide (binding energy (BE): ∼31.7 eV)^30^ and surface oxide (BE: ∼33.2 and ∼35.8 eV),^31^ but they also indicate a significant level of nitride (BE: ∼32.5 eV)^32^ and a small amount of ammonium salt (similar to the chemical shift of ammonium paratungstate: APT (BE: ∼36.9 eV)).^33^

The notable presence of nitride (BE: ∼397.4 eV)^32^ and ammonium (BE: ∼401.7 and ∼402.3 eV)^33,34^ is evident in Figure 4b,f at the N 1*s* peak, which also suggests the presence of amine and/or amide (BE: ∼399.9 eV).^35^ The presence of nitrile (BE: ∼400.0 eV)^35^ on the WC-AM powder surface is less plausible than amine and/or amide, which is elaborated in the TDS results.

The C 1*s* peaks in Figure 4c,g reveal a more significant signal associated with *sp*^2^-carbon (BE: ∼284.1 eV)^30^ than *sp*^3^-carbon (BE: ∼284.8 eV),^36^ which suggests the presence of a small quantity of graphitic carbon residues. The presence of amine (BE: ∼285.3 eV),^36^ ether/alcohol (BE: ∼286.2 eV),^36^ ketone/amide (BE: ∼287.7 eV),^36^ and urea (BE: ∼288.6 eV)^36^ is also evident.

The O 1*s* peaks in Figure 4d,h represent the presence of hydroxide/ketone (BE: ∼531.2 eV)^37,38^ and water (adsorbed or lattice water)/ether/alcohol (BE: ∼532.5 eV).^37,39^

### TaC Powders

The XPS spectra for the TaC-HCS and TaC-JNM powders differed significantly, which also aligns with their considerably different multiple HSPs as listed in Table 3.

Figure 5a,d show the chemical shifts of the Ta4 *f*_7/2_ peaks for the TaC-HCS and TaC-JNM powders, respectively. The Ta 4*f*_7/2_ peaks for TaC-HCS show the presence of Ta metal (BE: ∼21.9 eV),^40^ nitride (BE: ∼22.4 and ∼23.2 eV),^41^ carbide (BE: ∼23.6 eV),^42^ and surface oxide (BE: ∼24.7, ∼25.5, and ∼26.3 eV).^43,44^ The Ta 4*f*_7/2_ peaks for TaC-JNM indicate the likely presence of carbonate salt (BE: ∼27.8 eV), in addition to the absence of Ta metal and nitride. The presence of nitrile on both the TaC-HCS and TaC-JNM powder surfaces is less plausible while the presence of urea, amine, and/or amide is highly probable, as detailed later with the TDS results.

The C 1*s* peaks in Figure 5b,e reveal a more significant signal of *sp*^2^-carbon than *sp*^3^-carbon for the TaC-HCS powder, which suggests the presence of a small amount of graphitic carbon residues. In contrast, no *sp*^2^-carbon signal was observed for the TaC-JNM powder. The presence of amine, ether/alcohol, ketone/amide, and urea can be recognized for the TaC-HCS powder, while significant signals due to ether/alcohol, ester (BE: ∼288.6 eV),^36^ and carbonate salt (BE: ∼290.1 eV)^45^ were observed for the TaC-JNM powder.

The O 1*s* peaks in Figure 5c,f indicate the presence of hydroxide/ketone and water (adsorbed or lattice water)/ether/alcohol, and the absence of ester for the TaC-HCS powder. Conversely, for TaC-JNM powder, there is the presence of carbonate salt (BE: ∼531.6 eV),^46^ water (adsorbed or lattice water)/ether/alcohol, and ester (BE: ∼533.7 eV),^47^ in addition to the almost absence of hydroxide.

The presence of urea, amine, and amide for the TaC-HCS powder, and the presence of carbonate and ester and the absence of hydroxide for the TaC-JNM powder align well with the surface ligand identification from multiple HSPs in Table 3.

### Oxide Powders

The XPS spectra for WO_3_-AM and Ta_2_O_5_-KCL were essentially the same, with only minor differences in the surface ligand concentration, which correlates with their similar multiple HSPs as listed in Table 3. Figure 6a,e show the chemical shifts of the W 4*f*_7/2_ and Ta 4*f*_7/2_ peaks, which suggest the presence of oxide and the absence of carbide and nitride for both the WO_3_-AM and Ta_2_O_5_-KCL powders. A faint signal due to ammonium salt was also observed for WO_3_-AM. The N 1*s* peak in Figure 6b clearly indicates the presence of nitrile for the WO_3_-AM powder, as supported by the TDS results, irrespective of the absence of nitride in the W 4*f*_7/2_ peak. The C 1*s* peaks in Figure 6c,f show the presence of amine, nitrile, and urea, as well as the absence of ketone for both the WO_3_-AM and Ta_2_O_5_-KCL powders. The O 1*s* peaks in Figure 6d,g indicate the presence of only hydroxide, with almost no evidence of water/ether/alcohol and ester for both the WO_3_-AM and Ta_2_O_5_-KCL powders. It should be noted that the hydroxide signal for the Ta_2_O_5_-KCL powder was the weakest among all the test powders.

### TDS Analysis

**Figure 7:**
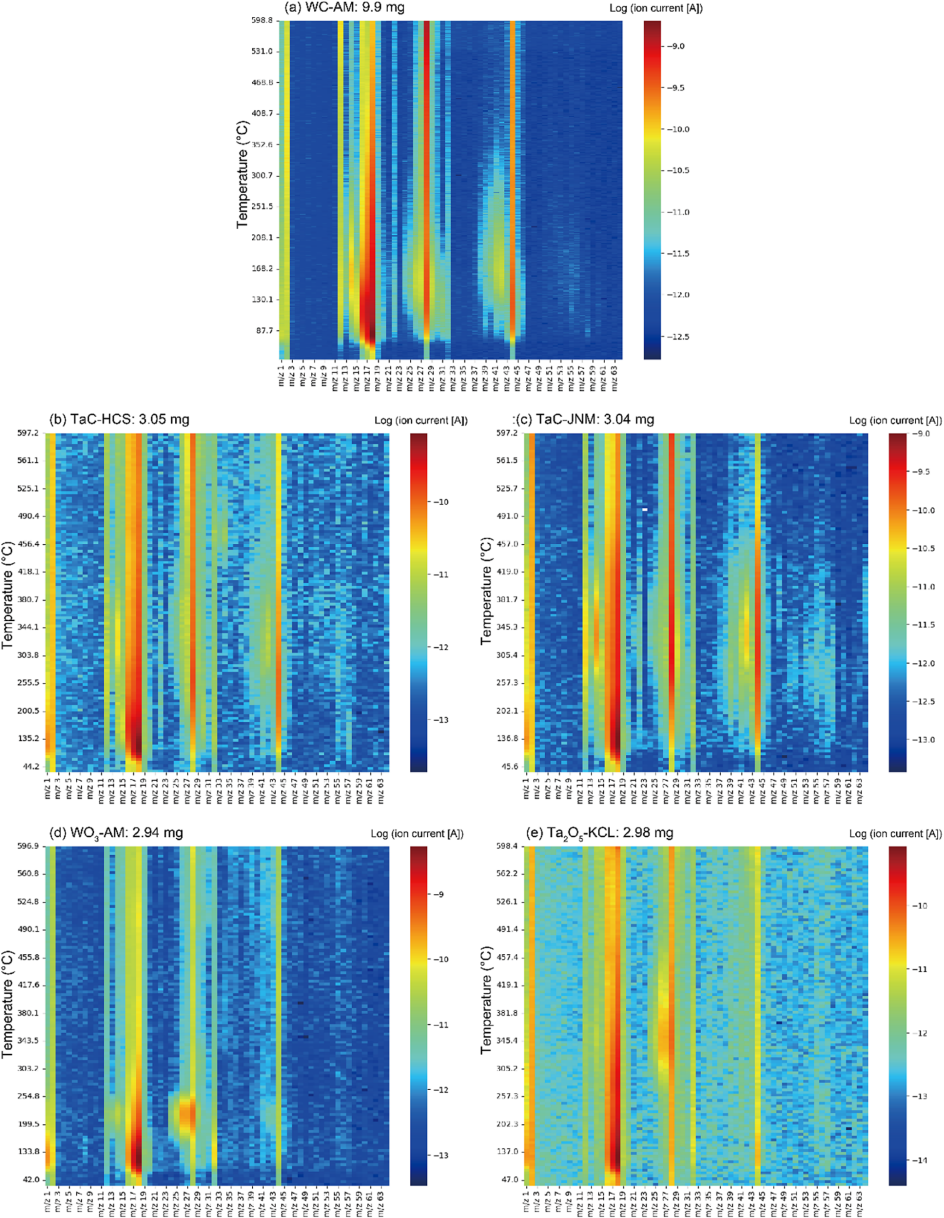
TDS spectra (*m*/*z* = 1–64) for test powders of (a) WC-AM, (b) TaC-HCS, (c) TaC-JNM, (d) WO_3_-AM, and (e) Ta_2_O_5_-KCL in the temperature range of RT–600 °C at a heating rate of 30 °C/min.

Figure 7 shows TDS spectra for the test powders, excluding the WC-HCS powder. All the spectra exhibited the strongest signals at *m*/*z* = 18 for H_2_O, 28 for CO, and 44 for CO_2_, due to adsorbed/absorbed water and various organic surface ligands. However, these are not particularly useful for the identification of surface ligands. In contrast, relatively weaker signals were observed at *m*/*z* = 17 for NH_3_, 27 for HCN, 30 for HCHO, and 42 for CH_2_CO, which suggests the presence of original surface ligands with respect to pyrolysis data,^48,49^ as listed in Table 4. The NH_3_ (*m*/*z*: 17) signal is strongly influenced by the presence of an OH fragment (*m*/*z*: 17) generated from H_2_O. Authentic NH_3_ signals were obtained by employing the formula (signal intensity of *m*/*z*: 17) – 0.406 × (signal intensity of *m*/*z*: 18) to eliminate the contribution from the OH fragment.

**Table 4:** Molecules Detected in TDS Analysis and their Origins (Surface Ligands) Suggested from Literature on Pyrolysis.^48,49^

*m*/*z*	detected molecule	original surface ligand suggested from literature on pyrolysis
17	Ammonia (NH_3_)	Ammonium salt, urea, amide, and/or primary amine
18	Water (H_2_O)	Hydroxide, adsorbed water, lattice water, and/or many organic ligands
27	Hydrogen cyanide (HCN)	Nitrile, amide, secondary/tertiary amine
28	Carbon monoxide (CO)	Many organic ligands
30	Formaldehyde (HCHO)	Ether and/or alcohol
42	Ketene (CH_2_CO)	Ketone
44	Carbon dioxide (CO_2_)	Carbonate salt and/or many organic ligands

**Figure 8:**
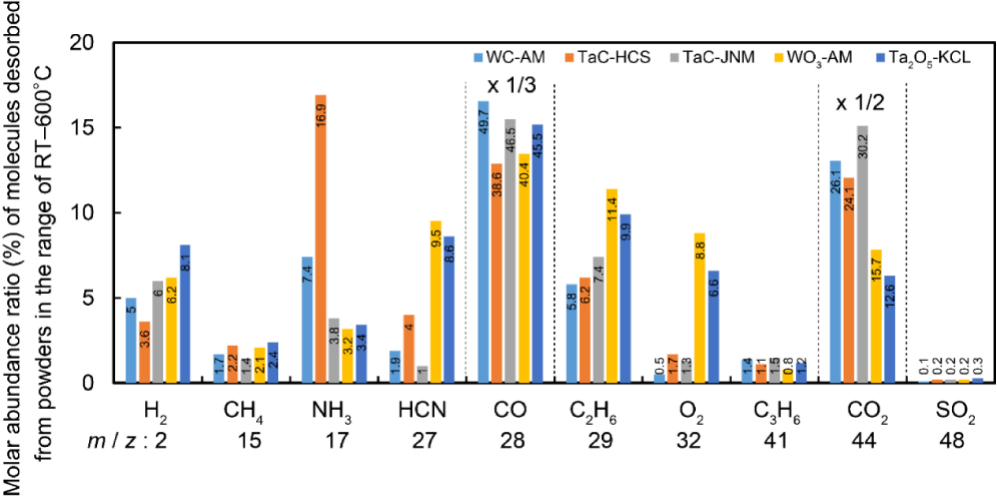
Summary of quantitative TDS results representing molar abundance ratio for molecules (except water) desorbed from powders, which were integrated in the temperature range of RT–600 °C. The bar heights for CO and CO_2_ were multiplied by factors of 1/3 and 1/2, respectively.

Quantitative TDS results are given in Figure 8. The WC-AM and TaC-HCS powders exhibited significant desorption of NH_3_, which suggests the presence of ammonium salt, urea, amide, and/or primary amine as surface ligands. Conversely, the WO_3_-AM and Ta_2_O_5_-KCL powders exhibited desorption of HCN, which implies the presence of nitrile, amide, and/or secondary/tertiary amine as surface ligands. Furthermore, the quantity of original surface ligands to form NH_3_ or HCN, as estimated from quantitative TDS results, represents 10–20% of surface atoms (with a reference areal atomic density of powder surfaces at approximately 2 × 10^15^ atoms/cm^2^), which suggests substantial portions of the powder surfaces (except TaC-JNM) are covered with nitrogen-related surface ligands.

The ranking of the TDS signal intensities for NH_3_, HCN, HCHO, and CH_2_CO (for which quantitative results are not available due to the absence of conversion factors for these molecules) derived from the test powders (including CO_2_ for the TaC-JNM powder) are given in Table 5. These rankings validate the surface ligands identified through XPS and HSP analyses.

**Table 5:** Summary of Molecules and their Original Surface Ligands Detected in TDS, XPS, and HSP Analyses, Comparison of the Rank in Signal Intensity for Molecules Detected in the TDS Analysis to Rank in Atomic Concentration for Possible Ligands Detected in the XPS Analysis, and to Rank the Coverage for Corresponding Top-Most Surface Ligands Identified from Multi-HSPs Analysis

	TDS		XPS		HSP	
test powder	rank in intensity	molecule detected in TDS	rank in atomic concentration^a^	ligand detected in XPS	rank in coverage	possible topmost surface ligand identified from multiple HSPs
WC-AM	#1	Ammonia (NH_3_: 1.2 × 10^–8^ C/mg)	#1 (<9.7 at%)	Ammonium salt (N: 0.7%, W: 1.7%), urea (N: <0.5%, C: 1.1%), amide (N: <0.5%, C: <1.3%), and primary amine (N: <0.5%, C: <3.9%)	#1 (36.2%)	Ammonium salt + urea + amine
	#2	Hydrogen cyanide (HCN: 4.0 × 10^–9^ C/mg)	#2 (<6.2 at%)	Amide (N: <0.5%, C: <1.3%) and secondary/tertiary amine (N: <0.5%, C: <3.9%)	#2 (32.7%)	Ketone + amide
	#3	Ketene (CH_2_CO: 2.0 × 10^–9^ C/mg)	#4 (<2.6 at%)	Ketone (C: <1.3%, O: ≪8.0%)		
	#4	Formaldehyde (HCHO: 8.8 × 10^–10^ C/mg)	#3 (<5.0 at%)	Ether and/or alcohol (C: 2.5%, O: ≪4.2%)	#3 (20.5%)	Ether and/or alcohol
WC-HCS			#1 (<7.6 at%)	Ammonium salt (N: 0.7%, W: 1.2%), urea (N: <0.4%, C: 1.1%), amide (N: <0.4%, C: <1.0 at%), and primary amine (N: <0.4%, C: <3.2%)	#1 (53.8%)	Hydroxide + ammonium salt + urea + amine
			#2 (<4.7 at%)	Amide (N: <0.4%, C: <1.1%) and secondary/tertiary amine (N: <0.4%, C: <3.2%)	#2 (29.4%)	Ketone + amide
			#4 (<2.0 at%)	Ketone (C: <1.0%, O: ≪10.7%)		
			#3 (<3.8 at%)	Ether and/or alcohol (C: 1.9%, O: ≪4.0%)	#3 (16.1%)	Ether and/or alcohol
TaC-HCS	#1	Ammonia (NH_3_: 1.3 × 10^–8^ C/mg)	#1 (<8.0 at%)	Urea (C: 1.8%), amide (C: <1.6%), and primary amine (C: <4.6%)	#4 (16.1%)	Urea + amine
	#2	Hydrogen cyanide (HCN: 4.1 × 10^–9^ C/mg)	#2 (<6.2 at%)	Amide (C: <1.6%) and secondary/tertiary amine (C: <4.6%)	#3 (24.0%)	Ketone + amide
	#3	Formaldehyde (HCHO: 1.1 × 10^–9^ C/mg)	#3 (<5.3 at%)	Ether and/or alcohol (C: 2.1%, O: <3.2%)	#1 (32.9%)	Ether and/or alcohol
	#4	Ketene (CH_2_CO: 9.9 × 10^–10^ C/mg)	#4 (<3.2 at%)	Ketone (C: <1.6%, O: ≪9.2%)	#3 (24.0%)	Ketone + amide
TaC-JNM	#1	Carbon dioxide (CO_2_: 4.0 × 10^–8^ C/mg)	#1 (19.8 at%)	Carbonate salt (C: 2.6%, O: 13.0%, Ta: 4.2%)	#1 (53.4%)	Carbonate salt
	#2	Ketene (CH_2_CO: 3.7 × 10^–9^ C/mg)	#3 (<11.1 at%)	Ketone (C: 1.8%, O <1.0%), ester (C: 4.4%, 3.9%)	#2 (33.9%)	Ester + ketone
	#3	Formaldehyde (HCHO: 9.6 × 10^–10^ C/mg)	#2 (<16.4 at%)	Ether and/or alcohol (C: 8.2%, O: <8.4%)	#3 (7.7%)	Ether and/or alcohol
WO_3_-AM	#1	Hydrogen cyanide (HCN: 8.9 × 10^–9^ C/mg)	#1 (<3.6 at%)	Nitrile (N: <1.2%, C: <2.0%), secondary/tertiary amine (N: <1.2%, C: <0.4%)	#1 (64.1%)	Nitrile
	#2	Ammonia, (NH_3_: 2.3 × 10^–9^ C/mg)	#2 (<1.9 at%)	Urea (N: <1.2%, C: 0.3%) and primary amine (N: <1.2%, C: <0.4%)	#2 (24.7%)	Hydroxide + urea + amine
	#3	Formaldehyde (HCHO: 1.3 × 10^–9^ C/mg)	#3 (∼0 at%)	Ether and/or alcohol (C: <2.0%, O: ∼0%)	#3 (11.2%)	Ether and/or alcohol
	#4	Ketene (CH_2_CO: 5.0 × 10^–10^ C/mg)	#3 (∼0 at%)	Ketone (C: ∼0%, O: ≪12.5%)	#5 (0.002%)	Anhydride
Ta_2_O_5_-KCL	#1	Hydrogen cyanide (HCN: 2.6 × 10^–9^ C/mg)	#1 (<3.7 at%)	Nitrile (C: <1.9%) and secondary/tertiary amine (C: <1.8%)	#2 (40.8%)	Nitrile
	#2	Ammonia (NH_3_: 7.8 × 10^–10^ C/mg)	#2 (<2.8 at%)	Urea (C: 1.0%) and primary amine (C: <1.8%)	#1 (41.8%)	Urea + amine
	#3	Formaldehyde (HCHO: 3.8 × 10^–10^ C/mg)	#3 (∼0 at%)	Ether and/or alcohol (C: <1.9%, O: ∼0%)	#3 (10.6%)	Ether and/or alcohol

a Atomic concentrations of oxygen for ether (and/or alcohol) and ketone in XPS are overestimated by the presence of hydroxide and water; therefore, the true concentration was assumed to be same as the counter-carbon concentration.

### Comparison Between TDS, XPS, and HSP Results

Table 5 also lists the rankings of atomic concentrations of surface ligands as detected by XPS, which can potentially form the desorbed-gas molecules identified in the TDS analysis. Table 5 also shows the ranking of corresponding surface ligand coverages estimated from the multi-HSP analysis. A comparison of the rankings from the TDS and XPS analyses reveals they are almost identical, which suggests that both the TDS and XPS results accurately reflect the types of surface ligands (excluding hydroxides) and their quantities. This suggests that the TDS and XPS analyses complementarily contribute to ensure the reliability of the identification of surface ligands on powder surfaces, which makes the identification and quantification of the surface ligands through TDS and XPS results highly credible.

In addition, the ranking based on coverage, as estimated from multi-HSP analysis, are in alignment with both the TDS and XPS results. A comparison between the XPS and HSP results on hydroxide concentrations, as presented in Table 6, also shows good agreement. This leads to the conclusion that multi-HSP analysis accurately reflects the types and quantities of surface ligands. The dispersion stability of powders is thus governed by the interaction of single/multiple surface ligands with relatively higher energies (higher HSP values) that cover powder surfaces. This confirms the utility of the proposed multi-HSP derivation method, which is invaluable for real-world formulators who seek to understand and control powder-related manufacturing processes.

**Table 6:** Comparison Between XPS and HSP Results for Hydroxide Concentrations

	XPS		HSP		
test powder	rank in atomic concentration	atomic concentration for oxygen assigned to M–OH and/or C=O (at%)	rank in coverage	top-most surface ligand coverage identified as hydroxide-related (%)	remark (identified complex ligands)
WO_3_-AM	1	12.5	3	<24.7	Hydroxide + urea + amine
WC-HCS	2	10.7	1	<53.8	Hydroxide + ammonium salt + urea + amine
TaC-HCS	3	9.2	2	27	Hydroxide
WC-AM	4	8.0	4	10.6	Hydroxide
Ta_2_O_5_-KCL	5	5.8	5	7.5	Hydroxide
TaC-JNM	6	1.0	6	∼0	No hydroxide

Furthermore, dispersion stability measurements combined with multi-HSP analysis should be considered as the most surface-sensitive technique in powder-surface analysis. This is because probe liquid molecules directly touch/interact with surface ligands to allow the detection of their affinity and interaction. This sensitivity extends only to the topmost surface ligands. In contrast, XPS analysis of powder surfaces typically includes information not only on the topmost surface but also on subsurface layers, with an information depth of approximately 3 nm.^40^ Furthermore, TDS analysis additionally provides bulk information, such as that on absorbed water. Both XPS and TDS can thus be classified as semisurface-sensitive techniques. Consequently, when it comes to characterizing powder surfaces, dispersion stability measurements paired with multi-HSP analysis are superior to conventional XPS and TDS analyses in terms of both topmost surface sensitivity and practicality.

## Conclusions

This study assessed the dispersion stability of industrial carbide/oxide powders within the HSP framework. The conventional single-sphere fitting method and HSPs derived by this method partially succeed in accounting for the surface ligands that dominate dispersion stability. However, the proposed log-fit approach, bolstered by multi-HSP analysis, significantly improved the fit of the experimental results for various mosaic-surface powders with complex surface ligands. XPS and TDS analyses were effectively utilized in conjunction to decipher the surface ligands on these mosaic-surface powders, which facilitated credible identification and quantification of the surface ligands. These results align well with the surface ligands and their coverage as determined by the multi-HSP analysis. It is concluded that multi-HSP analysis accurately reflects the types and quantities of topmost surface ligands on powder surfaces. The dispersion stability of powders is governed by the interaction of single/multiple surface ligands with higher energies (higher-HSP values) that cover powder surfaces. The proposed method represents a valid contribution to the understanding and control of powder-related research and manufacturing processes.

## Supporting Information Available

SEM image of sample powders, particle-diameter distribution by laser diffraction measurement, list of probe liquids and their HSPs, photographs of dispersions, summary of experimental and data-fitting results for relative sedimentation time for powders, and summary of chemical bonds/surface ligands and their concentrations by XPS analyses (PDF)
