# Supplementary material for: Dispersion Stability of Inorganic Powders Harnessed to Mosaic Surface Ligands via Multifit Hansen Solubility Parameters
Source: Langmuir. 2024 Jul 15;40(29):14823–37. doi: 10.1021/acs.langmuir.4c00641 (PMC11270992; doi:10.1021/acs.langmuir.4c00641)
Supplement: Supplementary file 1 — la4c00641_si_001.pdf [file la4c00641_si_001.pdf]

Supporting information for

Dispersion stability of inorganic powders harnessed to mosaic surface ligands via multifit Hansen solubility parameters

Daisuke Nakamura[1,*] and Naoko Takahashi[1]

[1]*Toyota Central R&D Laboratories, Inc., Nagakute, Aichi 480-1192, Japan*

*E-mail: daisuke@mosk.tytlabs.co.jp

Table of Contents

Figure S1 SEM images of as-received sample powders……………….…………………………S2

Figure S2 Particle size distributions measured by laser diffraction method…………………………S3

Table S1 Probe liquids for WC and WO_3 test powders……………………………………………...S4

Table S2 Probe liquids for TaC and Ta_2O_5 test powders…………………………………………S5

Table S3 Sedimentation time for WC-AM powder……………………………………………………S6

Table S4 Sedimentation time for WC-HCS powder…………………………………………………S7

Table S5 Sedimentation time for TaC-HCS powder…………………………………………………S8

Table S6 Sedimentation time for TaC-JNM powder…………………………………………….....S9

Table S7 Sedimentation time for WO_3-AM powder…………………………………………...S10

Table S8 Sedimentation time for Ta_2O_5-KCL powder……………………………………….…...S11

Figure S3 Photographs of dispersions of sample powders……..……………………………S12

Figure S4 Pseudo-3D HSP plots of test solvents for TaC powders…………………………………S13

Figure S5 Pseudo-3D HSP plots of test solvents for Oxide powders……………………………..S14

Figure S6 Correlation of log (*RST*) to harmonic-mean HSP distance………………………………S15

Table S9 Chemical bonds/surface ligands identified in XPS spectra from test powders…………..S16

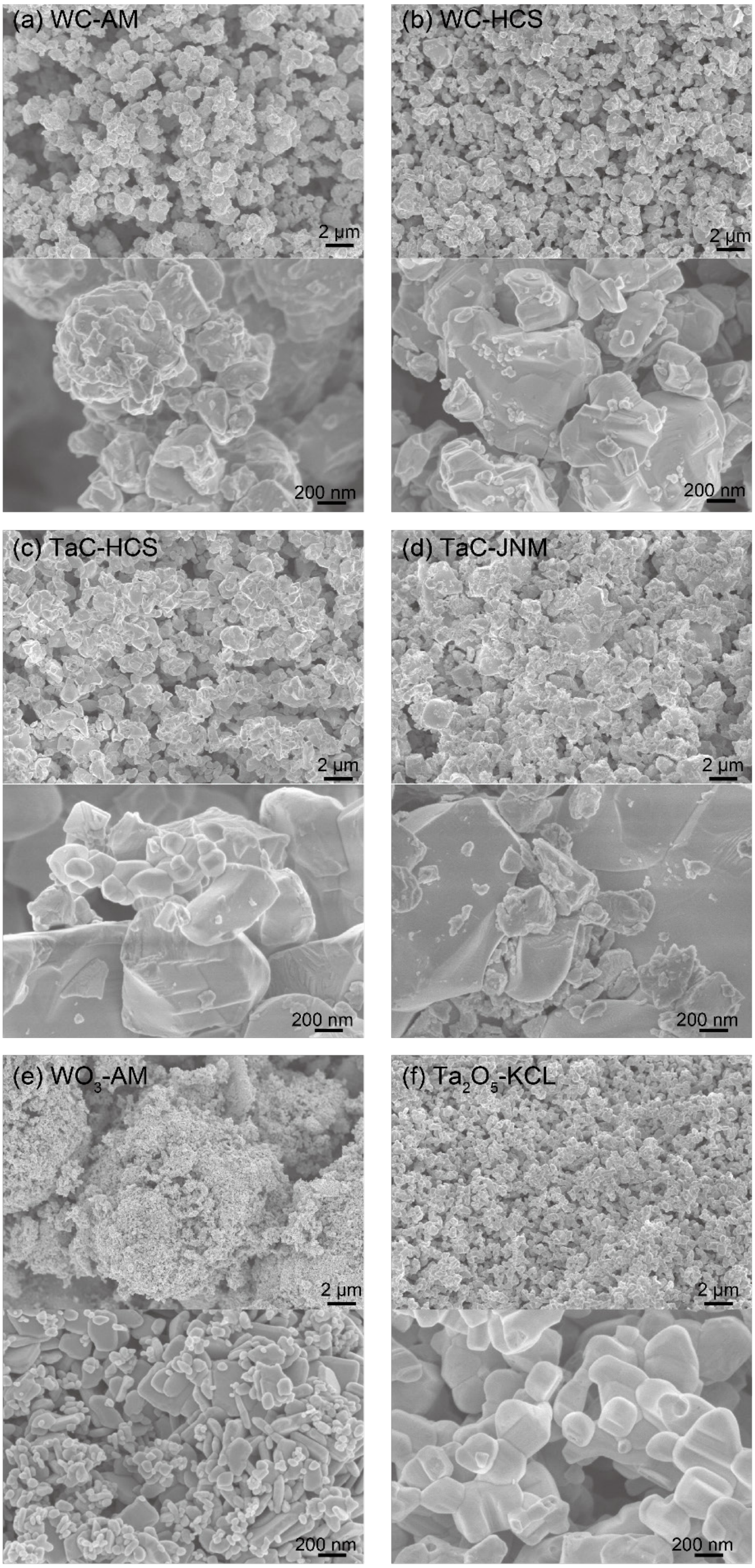

Fig. S1 Low magnification (top) and high magnification (bottom) SEM images of as-received (a) WC-AM, (b) WC-HCS, (c) TaC-HCS, (d) TaC-JNM, (e) WO_3-AM, and (f) Ta_2O_5-KCL powders.

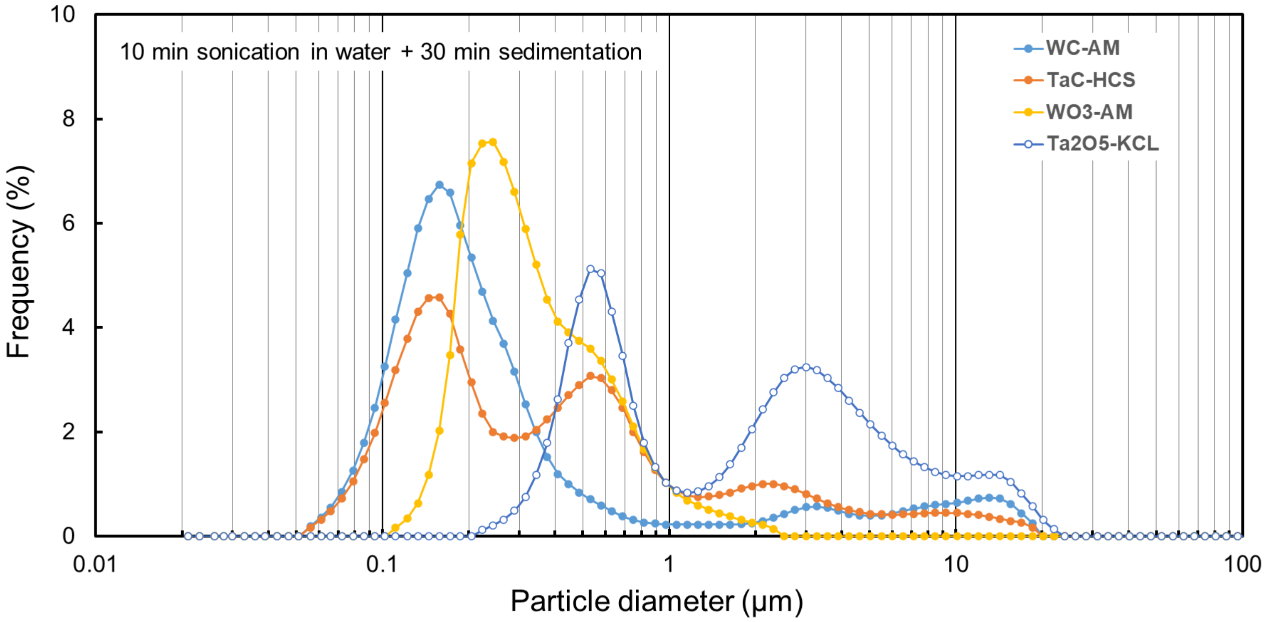

Fig. S2 Particle size distributions measured by laser diffraction method for WC-AM, TaC-HCS, WO_3-AM, and Ta_2O_5-KCL powders in water dispersions. The sample dispersions were taken from opaque supernatant layers after 10 min sonication in ultrasonic cleaning bath and subsequent 30 min sedimentation.

Table S1 Probe liquids for WC and WO_3 test powders as well as their HSPs, density, and viscosity values.

No.	Probe liquid	δD ($[J/cm^3]^{1/2}$)	δP ($[J/cm^3]^{1/2}$)	δH ($[J/cm^3]^{1/2}$)	Density of probe liquid, ρ_L (g/cm^3)	Viscosity of probe liquid, η (mPa・sec)
#1	Dimethyl Carbonate	15.5	8.6	9.7	1.069	0.584
#2	Diethylene Glycol Monobutyl Ether	16	7	10.6	0.952	5.052
#3	Diethylene Glycol Monoethyl Ether	16.1	9.2	12.2	0.984	3.828
#4	Acetone	15.5	10.4	7	0.79	0.312
#5	Diethylene Glycol Monomethyl Ether	16.2	7.8	12.6	1.017	3.565
#6	Ethylene Glycol Monoethyl Ether	15.9	7.2	14	0.93	1.838
#7	Methyl Ethyl Ketone (MEK)	16	9	5.1	0.805	0.378
#8	Ethylene Glycol Monomethyl Ether	16	8.2	15	0.96	1.541
#9	n-Butyl Acetate	15.8	3.7	6.3	0.87	0.678
#10	1-Butanol	16	5.7	15.8	0.81	2.619
#11	Diethyl Ether	14.5	2.9	4.6	0.72	0.224
#12	2-Propanol	15.8	6.1	16.4	0.781	2.015
#13	1,3-Dioxolane	18.1	6.6	9.3	1.065	0.589
#14	1-Nitropropane	16.6	12.3	5.5	0.998	0.798
#15	Glycerol	17.4	11.3	27.2	1.257	964
#16	Dimethyl Acetamide (DMA)	16.8	11.5	9.4	0.937	0.945
#17	1,4-Dioxane	17.5	1.8	9	1.04	1.196
#18	Cyclohexanone	17.8	8.4	5.1	0.947	2.02
#19	Caprolactone (Epsilon)	18	15	7.4	1.067	5.532
#20	Dipropylene Glycol	16.5	10.6	17.7	1.0206	79.06
#21	Ethanol	15.8	8.8	19.4	0.82	1.082
#22	Acetonitrile	15.3	18	6.1	0.786	0.342
#23	Dimethyl Sulfoxide (DMSO)	18.4	16.4	10.2	1.1	1.991
#24	2-Chlorophenol	19	5.5	13.9	1.241	3.376
#25	Formamide	17.2	26.2	19	1.13	3.322
#26	Propylene Carbonate	20	18	4.1	1.188	2.5
#27	γ-Butyrolactone (GBL)	18	16.6	7.4	1.125	1.745
#28	N-Methyl-2-Pyrrolidone (NMP)	18	12.3	7.2	1.025	1.695
#29	Thiazole	20.5	18.8	10.8	1.2	1.007
#30	N-Methyl Formamide	17.4	18.8	15.9	1.011	1.65
#31	Diethylene Glycol	16.6	12	19	1.114	27.15
#32	Ethylenediamine	16.6	8.8	17	0.893	1.082
#33	Ethanolamine	17	15.5	21	1.014	19.342
#34	Benzyl Alcohol	18.4	6.3	13.7	1.045	5.376
#35	Ethylene Glycol	17	11	26	1.113	16.3876
#36	Formic Acid	14.6	10	14	1.214	1.51
#37	Acetic Acid	14.5	8	13.5	1.049	1.115
#38	Acetic Anhydride	16	11.7	10.2	1.082	0.842
#39	Water (1% Soluble In - Ro=18.1)	15.1	20.4	16.5	0.998	0.89
#40	1.3-Butanediol	16.5	8.1	20.9	1.005	97.262
#41	Dimethyl Formamide	17.4	13.7	11.3	0.944	0.8006
#42	Acetylacetone	16.1	10	6.2	0.957	0.737
#43	Benzonitrile	18.8	12	3.3	1.01	1.238
#44	Methanol	14.7	12.3	22.3	0.791	0.55
#45	2-Butanol	15.8	5.7	14.5	0.806	3.08
#46	Isobutyl Alcohol	15.1	5.7	15.9	0.802	3.389
#47	1-Hexanol	15.9	5.8	12.5	0.819	4.59
#48	Cyclohexanol	17.4	4.1	13.5	0.963	56.5
#49	Salicylaldehyde	19	10.5	12	1.146	2.501

Table S2 Probe liquids for TaC and Ta_2O_5 test powders as well as their HSPs, density, and viscosity values.

No.	Probe liquid	δD ([J/cm^3]$^{1/2}$)	δP ([J/cm^3]$^{1/2}$)	δH ([J/cm^3]$^{1/2}$)	Density of probe liquid, ρ_L (g/cm^3)	Viscosity of probe liquid, η (mPa・sec)
#1	Dimethyl Carbonate	15.5	8.6	9.7	1.069	0.584
#2	Diethylene Glycol Monobutyl Ether	16	7	10.6	0.952	5.052
#3	Diethylene Glycol Monoethyl Ether	16.1	9.2	12.2	0.984	3.828
#4	Acetone	15.5	10.4	7	0.79	0.312
#5	Diethylene Glycol Monomethyl Ether	16.2	7.8	12.6	1.017	3.565
#6	Ethylene Glycol Monoethyl Ether	15.9	7.2	14	0.93	1.838
#7	Methyl Ethyl Ketone (MEK)	16	9	5.1	0.805	0.378
#8	Ethylene Glycol Monomethyl Ether	16	8.2	15	0.96	1.541
#9	n-Butyl Acetate	15.8	3.7	6.3	0.87	0.678
#10	1-Butanol	16	5.7	15.8	0.81	2.619
#11	Propylene Carbonate	20	18	4.1	1.188	2.5
#12	2-Propanol	15.8	6.1	16.4	0.781	2.015
#13	1,3-Dioxolane	18.1	6.6	9.3	1.065	0.589
#14	1-Nitropropane	16.6	12.3	5.5	0.998	0.798
#15	Glycerol	17.4	11.3	27.2	1.257	964
#16	Dimethyl Acetamide (DMA)	16.8	11.5	9.4	0.937	0.945
#17	1,4-Dioxane	17.5	1.8	9	1.04	1.196
#18	Cyclohexanone	17.8	8.4	5.1	0.947	2.02
#19	Caprolactone (Epsilon)	18	15	7.4	1.067	5.532
#20	Dipropylene Glycol	16.5	10.6	17.7	1.0206	79.06
#21	Ethanol	15.8	8.8	19.4	0.82	1.082
#22	Acetonitrile	15.3	18	6.1	0.786	0.342
#23	Dimethyl Sulfoxide (DMSO)	18.4	16.4	10.2	1.1	1.991
#24	2-Chlorophenol	19	5.5	13.9	1.241	3.376
#25	Formamide	17.2	26.2	19	1.13	3.322
#26	N-Methyl Formamide	17.4	18.8	15.9	1.011	1.65
#27	γ-Butyrolactone (GBL)	18	16.6	7.4	1.125	1.745
#28	N-Methyl-2-Pyrrolidone (NMP)	18	12.3	7.2	1.025	1.695
#29	Dimethyl Formamide	17.4	13.7	11.3	0.944	0.8006
#30	Methanol	14.7	12.3	22.3	0.791	0.55
#31	Ethylene Glycol	17	11	26	1.113	16.3876
#32	1,3-Butanediol	16.5	8.1	20.9	1.005	97.262
#33	Diethylene Glycol	16.6	12	19	1.114	27.15
#34	Cyclohexanol	17.4	4.1	13.5	0.963	56.5
#35	Benzyl Alcohol	18.4	6.3	13.7	1.045	5.376
#36	Acetic Anhydride	16	11.7	10.2	1.082	0.842
#37	Thiazole	20.5	18.8	10.8	1.2	1.007
#38	Pyridine	19	8.8	5.9	0.978	1.227
#39	Nitrobenzene	20	10.6	3.1	1.196	1.686
#40	Water (1% Soluble In - Ro=18.1)	15.1	20.4	16.5	0.998	0.89
#41	Methylene Diiodide (Diiodomethane)	22	3.9	5.5	3.325	2.644
#42	Acetic Acid	14.5	8	13.5	1.049	1.115
#43	Aniline	20.1	5.8	11.2	1.022	3.69
#44	Formic Acid	14.6	10	14	1.214	1.51
#45	Salicylaldehyde	19	10.5	12	1.146	2.501
#46	Dichloroacetic Acid	18.2	8.1	12.2	1.553	5.06
#47	Acetol	17.6	12.7	16.4	1.042	4.395
#48	Ethanolamine	17	15.5	21	1.014	19.342
#49	Ethylenediamine	16.6	8.8	17	0.893	1.082
#50	Thiophenol	20	4.5	10.3	1.073	1.144

Table S3 Sedimentation time for 3.0 g of WC-AM powder in dispersions with 2 mL each of the probe liquids, relative sedimentation time, dispersion stability, and relative energy difference.

No.	Probe liquid	Sedimentation time, t_{sed} (sec)	Relative sedimentation time, *RST* (arb. Unit)	Dispersion stability (1: good, 0: poor)†	Relative energy difference, *RED*
#1	Dimethyl Carbonate	180	4294	0	1.142
#2	Diethylene Glycol Monobutyl Ether	240	667	0	1.21
#3	Diethylene Glycol Monoethyl Ether	240	879	0	0.99*
#4	Acetone	20	911	0	1.191
#5	Diethylene Glycol Monomethyl Ether	240	941	0	1.077
#6	Ethylene Glycol Monoethyl Ether	60	459	0	1.076
#7	Methyl Ethyl Ketone (MEK)	30	1127	0	1.386
#8	Ethylene Glycol Monomethyl Ether	90	820	0	0.974*
#9	n-Butyl Acetate	60	1250	0	1.63
#10	1-Butanol	61680	334188	1	1.159*
#11	Diethyl Ether	20	1275	0	1.776
#12	2-Propanol	950400	6706570	1	1.117*
#13	1,3-Dioxolane	80	1893	0	1.358
#14	1-Nitropropane	240	4211	0	1.215
#15	Glycerol	13812000	196907	1	1.045*
#16	Dimethyl Acetamide (DMA)	120	1786	0	1.003
#17	1,4-Dioxane	60	700	0	1.674
#18	Cyclohexanone	60	417	0	1.461
#19	Caprolactone (Epsilon)	160080	403181	1	1.043*
#20	Dipropylene Glycol	950400	168050	1	0.754
#21	Ethanol	110	1442	0	0.891*
#22	Acetonitrile	58020	2411393	1	1.005*
#23	Dimethyl Sulfoxide (DMSO)	80	559	0	0.847*
#24	2-Chlorophenol	100	408	0	1.333
#25	Formamide	1052760	4395479	1	0.606
#26	Propylene Carbonate	2591600	14318072	1	1.373*
#27	γ-Butyrolactone (GBL)	2591600	20606562	1	1.004*
#28	N-Methyl-2-Pyrrolidone (NMP)	180	1484	0	1.155
#29	Thiazole	180	2467	0	1.013
#30	N-Methyl Formamide	1381200	11710065	1	0.36
#31	Diethylene Glycol	326400	166939	1	0.648
#32	Ethylenediamine	2580	33638	0‡	0.907
#33	Ethanolamine	8820	6378	0‡	0.467
#34	Benzyl Alcohol	660	1713	0	1.241
#35	Ethylene Glycol	235560	199616	1	0.978
#36	Formic Acid	960	8765	0	0.875*
#37	Acetic Acid	600	7507	0	1.042
#38	Acetic Anhydride	120360	1989514	1	0.927
#39	Water (1% Soluble In - Ro=18.1)	71760	1128970	1‡	0.176
#40	1.3-Butanediol	28800	4144	0	0.979*
#41	Dimethyl Formamide	120	2107	0	0.804*
#42	Acetylacetone	180	3430	0	1.265
#43	Benzonitrile	240	2712	0	1.472
#44	Methanol	180	4650	0	0.7*
#45	2-Butanol	1307220	6024247	1	1.181*
#46	Isobutyl Alcohol	1307220	5476515	1	1.158*
#47	1-Hexanol	252420	779862	1	1.226*
#48	Cyclohexanol	977820	242932	1	1.362*
#49	Salicylaldehyde	600	3324	0	1.064

† Threshold to categorize into good/poor solvents was set to *RST* = 100,000.

‡ The dispersion stability with water as well as discolored probe liquids was omitted from the dataset to calculate HSP via sphere fitting.

* Anomalies indicating poor solvents inside HSP sphere or good solvents outside HSP spehere.

Table S4 Sedimentation time for 3.0 g of WC-HCS powder in dispersions with 2 mL each of the probe liquids, relative sedimentation time, dispersion stability, and relative energy difference.

No.	Probe liquid	Sedimentation time, t_{sed} (sec)	Relative sedimentation time, *RST* (arb. Unit)	Dispersion stability (1: good, 0: poor)†	Relative energy difference, *RED*
#1	Dimethyl Carbonate	120	2863	0	1.053
#2	Diethylene Glycol Monobutyl Ether	780	2169	0	1.103
#3	Diethylene Glycol Monoethyl Ether	2280	8348	0	0.921*
#4	Acetone	480	21862	0	1.112
#5	Diethylene Glycol Monomethyl Ether	360	1412	0	0.985*
#6	Ethylene Glycol Monoethyl Ether	360	2756	0	0.967*
#7	Methyl Ethyl Ketone (MEK)	660	24785	0	1.28
#8	Ethylene Glycol Monomethyl Ether	1320	12026	0	0.879*
#9	n-Butyl Acetate	120	2501	0	1.46
#10	1-Butanol	49414	267731	1	1.017*
#11	Diethyl Ether	120	7650	0	1.574
#12	2-Propanol	69180	488174	1	0.976
#13	1,3-Dioxolane	120	2839	0	1.248
#14	1-Nitropropane	10	175	0	1.154
#15	Glycerol	9604886	136929	1	0.819
#16	Dimethyl Acetamide (DMA)	48300	718776	1	0.967
#17	1,4-Dioxane	300	3502	0	1.491
#18	Cyclohexanone	460	3200	0	1.354
#19	Caprolactone (Epsilon)	66360	167136	1	1.029*
#20	Dipropylene Glycol	497760	88014	1	0.683
#21	Ethanol	47400	621194	1	0.758
#22	Acetonitrile	13600	565235	1	0.979
#23	Dimethyl Sulfoxide (DMSO)	330	2304	0	0.868*
#24	2-Chlorophenol	600	2445	0	1.203
#25	Formamide	86700	361989	1	0.551
#26	Propylene Carbonate	64800	358007	1	1.313*
#27	γ-Butyrolactone (GBL)	64800	515244	1	1
#28	N-Methyl-2-Pyrrolidone (NMP)	46286	381618	1	1.112*
#29	Thiazole	120	1644	0	1.004
#30	N-Methyl Formamide	64800	549386	1	0.457
#31	Diethylene Glycol	415800	212663	1	0.582
#32	Ethylenediamine	1380	17992	0‡	0.812
#33	Ethanolamine	4200	3037	0‡	0.408
#34	Benzyl Alcohol	7800	20247	0	1.128
#35	Ethylene Glycol	332400	281679	1	0.768
#36	Formic Acid	39000	356062	1	0.792
#37	Acetic Acid	300	3754	0	0.927*
#38	Acetic Anhydride	9780	161660	1	0.893
#39	Water (1% Soluble In - Ro=18.1)	25200	396461	1‡	0.264
#40	1.3-Butanediol	414180	59596	1	0.824
#41	Dimethyl Formamide	120	2107	0	0.813*
#42	Acetylacetone	9540	181778	1‡	1.181
#43	Benzonitrile	2880	32545	0	1.382
#44	Methanol	9240	238711	1	0.526
#45	2-Butanol	15400	70970	1	1.044*
#46	Isobutyl Alcohol	23100	96776	1	1.005*
#47	1-Hexanol	250440	773745	1	1.099*
#48	Cyclohexanol	413040	102617	1	1.213*
#49	Salicylaldehyde	420	2327	0	1.015

† Threshold to categorize into good/poor solvents was set to *RST* = 50,000.

‡ The dispersion stability with water as well as discolored probe liquids was omitted from the dataset to calculate HSP via sphere fitting.

* Anomalies indicating poor solvents inside HSP sphere or good solvents outside HSP spehere.

Table S5 Sedimentation time for 3.0 g of TaC-HCS powder in dispersions with 2 mL each of the probe liquids, relative sedimentation time, dispersion stability, and relative energy difference.

No.	Probe liquid	Sedimentation time, t_{sed} (sec)	Relative sedimentation time, *RST* (arb. Unit)	Dispersion stability (1: good, 0: poor)†	Relative energy difference, *RED*
#1	Dimethyl Carbonate	210	4650	0	1.318
#2	Diethylene Glycol Monobutyl Ether	1560	4029	0	1.276
#3	Diethylene Glycol Monoethyl Ether	1200	4080	0	1.116
#4	Acetone	110	4657	0	1.443
#5	Diethylene Glycol Monomethyl Ether	2100	7648	0	1.128
#6	Ethylene Glycol Monoethyl Ether	660	4693	0	1.101
#7	Methyl Ethyl Ketone (MEK)	180	6283	0	1.556
#8	Ethylene Glycol Monomethyl Ether	480	4062	0	1.01
#9	n-Butyl Acetate	300	5810	0	1.642
#10	1-Butanol	30000	151088	1	1.074*
#11	Propylene Carbonate	191400	980887	1	1.463*
#12	2-Propanol	191400	1255641	1	1.049*
#13	1,3-Dioxolane	180	3953	0	1.257
#14	1-Nitropropane	210	3422	0	1.45
#15	Glycerol	6811500	90040	1	0.526
#16	Dimethyl Acetamide (DMA)	270	3732	0	1.191
#17	1,4-Dioxane	270	2926	0	1.49
#18	Cyclohexanone	570	3683	0	1.487
#19	Caprolactone (Epsilon)	339300	793233	1	1.246*
#20	Dipropylene Glycol	773100	126921	1	0.765
#21	Ethanol	150600	1834481	1	0.837
#22	Acetonitrile	90	3477	0	1.497
#23	Dimethyl Sulfoxide (DMSO)	600	3887	0	1.041
#24	2-Chlorophenol	690	2608	0	1.009
#25	Formamide	420	1627	0	1.002
#26	N-Methyl Formamide	303000	2385253	1	0.771
#27	γ-Butyrolactone (GBL)	152400	1124441	1	1.253*
#28	N-Methyl-2-Pyrrolidone (NMP)	300	2296	0	1.272
#29	Dimethyl Formamide	210	3425	0	1.009
#30	Methanol	66400	1594687	1	0.822
#31	Ethylene Glycol	426720	335567	1	0.553
#32	1,3-Butanediol	696720	93087	1	0.754
#33	Diethylene Glycol	426720	202531	1	0.664
#34	Cyclohexanol	336720	77696	1	1.158*
#35	Benzyl Alcohol	1020	2458	0	1.001
#36	Acetic Anhydride	1920	29457	0	1.191
#37	Thiazole	510	6483	0	1.003
#38	Pyridine	1300	13797	0	1.393
#39	Nitrobenzene	540	4101	0	1.547
#40	Water (1% Soluble In - Ro=18.1)	79320	1158785	1‡	1.002
#41	Methylene Diiodide (Diiodomethane)	10	40	0	1.579
#42	Acetic Acid	300	3485	0	1.224
#43	Aniline	780	2743	0	1.134
#44	Formic Acid	540	4572	0	1.14
#45	Salicylaldehyde	3720	19119	0	0.940*
#46	Dichloroacetic Acid	1860	4575	0	1.025
#47	Acetol	780	2300	0	0.683*
#48	Ethanolamine	2100	1410	0‡	0.512
#49	Ethylenediamine	210	2544	0‡	0.845
#50	Thiophenol	360	4068	0	1.243

† Threshold to categorize into good/poor solvents was set to *RST* = 50,000.

‡ The dispersion stability with water as well as discolored probe liquids was omitted from the dataset to calculate HSP via sphere fitting.

* Anomalies indicating poor solvents inside HSP sphere or good solvents outside HSP spehere.

Table S6 Sedimentation time for 3.0 g of TaC-JNM powder in dispersions with 2 mL each of the probe liquids, relative sedimentation time, dispersion stability, and relative energy difference.

No.	Probe liquid	Sedimentation time, t_{sed} (sec)	Relative sedimentation time, *RST* (arb. Unit)	Dispersion stability (1: good, 0: poor)†	Relative energy difference, *RED*
#1	Dimethyl Carbonate	40	886	0	1.645
#2	Diethylene Glycol Monobutyl Ether	580	1498	0	1.754
#3	Diethylene Glycol Monoethyl Ether	390	1326	0	1.549
#4	Acetone	270	11432	0	1.52
#5	Diethylene Glycol Monomethyl Ether	520	1894	0	1.678
#6	Ethylene Glycol Monoethyl Ether	240	1707	0	1.78
#7	Methyl Ethyl Ketone (MEK)	50	1745	0	1.655
#8	Ethylene Glycol Monomethyl Ether	150	1269	0	1.705
#9	n-Butyl Acetate	60	1162	0	2.126
#10	1-Butanol	390	1964	0	1.958
#11	Propylene Carbonate	4071840	20867366	1	0.854
#12	2-Propanol	270	1771	0	1.952
#13	1,3-Dioxolane	70	1537	0	1.699
#14	1-Nitropropane	140	2281	0	1.306
#15	Glycerol	237600	3141	0	2.12
#16	Dimethyl Acetamide (DMA)	4740	65522	0	1.271
#17	1,4-Dioxane	160	1734	0	2.2
#18	Cyclohexanone	280	1809	0	1.614
#19	Caprolactone (Epsilon)	325500	760971	1	0.906
#20	Dipropylene Glycol	516300	84762	0	1.563
#21	Ethanol	180	2193	0	1.844
#22	Acetonitrile	2280	88093	0	1.016
#23	Dimethyl Sulfoxide (DMSO)	460	2980	0	0.697*
#24	2-Chlorophenol	330	1247	0	1.833
#25	Formamide	115800	448629	1	1
#26	N-Methyl Formamide	9053400	71269462	1	0.784
#27	γ-Butyrolactone (GBL)	115800	854398	1	0.76
#28	N-Methyl-2-Pyrrolidone (NMP)	64140	490983	1	1.169*
#29	Dimethyl Formamide	10628.57143	173328	0	1.019
#30	Methanol	60	1441	0	1.859
#31	Ethylene Glycol	1800	1415	0	2.055
#32	1,3-Butanediol	14475	1934	0	1.937
#33	Diethylene Glycol	3300	1566	0	1.515
#34	Cyclohexanol	433980	100138	0	1.992
#35	Benzyl Alcohol	630	1518	0	1.753
#36	Acetic Anhydride	3240	49708	0	1.315
#37	Thiazole	120	1525	0‡	0.54
#38	Pyridine	120	1274	0	1.531
#39	Nitrobenzene	2160	16404	0	1.493
#40	Water (1% Soluble In - Ro=18.1)	90	1315	0‡	1.045
#41	Methylene Diiodide (Diiodomethane)	10	40	0	2.114
#42	Acetic Acid	100	1162	0‡	1.821
#43	Aniline	2100	7386	0	1.783
#44	Formic Acid	250	2117	0‡	1.652
#45	Salicylaldehyde	330	1696	0‡	1.298
#46	Dichloroacetic Acid	870	2140	0‡	1.552
#47	Acetol	410	1209	0	1.259
#48	Ethanolamine	2700	1813	0‡	1.392
#49	Ethylenediamine	210	2544	0‡	1.681
#50	Thiophenol	190	2147	0	1.911

† Threshold to categorize into good/poor solvents was set to *RST* = 400,000.

‡ The dispersion stability with water as well as discolored probe liquids was omitted from the dataset to calculate HSP via sphere fitting.

* Anomalies indicating poor solvents inside HSP sphere or good solvents outside HSP spehere.

Table S7 Sedimentation time for 0.7 g of WO_3-AM powder in dispersions with 2 mL each of the probe liquids, relative sedimentation time, dispersion stability, and relative energy difference.

No.	Probe liquid	Sedimentation time, t_{sed} (sec)	Relative sedimentation time, *RST* (arb. Unit)	Dispersion stability (1: good, 0: poor)†	Relative energy difference, *RED*
#1	Dimethyl Carbonate	50	508	0	1.292
#2	Diethylene Glycol Monobutyl Ether	600	718	0	1.4
#3	Diethylene Glycol Monoethyl Ether	460	723	0	1.192
#4	Acetone	660	13137	0	1.214
#5	Diethylene Glycol Monomethyl Ether	360	604	0	1.313
#6	Ethylene Glycol Monoethyl Ether	910	3005	0	1.383
#7	Methyl Ethyl Ketone (MEK)	790	12947	0	1.38
#8	Ethylene Glycol Monomethyl Ether	780	3057	0	1.305
#9	n-Butyl Acetate	40	362	0	1.778
#10	1-Butanol	900	2127	0	1.537
#11	Diethyl Ether	3	84	0	1.945
#12	2-Propanol	570	1759	0	1.52
#13	1,3-Dioxolane	20	202	0	1.442
#14	1-Nitropropane	120	903	0	1.095
#15	Glycerol	28284750	168506	0	1.659
#16	Dimethyl Acetamide (DMA)	1080	6929	0	1.006
#17	1,4-Dioxane	150	747	0	1.866
#18	Cyclohexanone	120	360	0	1.411
#19	Caprolactone (Epsilon)	701400	752243	1	0.8
#20	Dipropylene Glycol	153300	11594	0	1.159
#21	Ethanol	240	1371	0	1.39
#22	Acetonitrile	33000	599596	1	0.772
#23	Dimethyl Sulfoxide (DMSO)	2640	7823	0	0.593*
#24	2-Chlorophenol	2280	3889	0	1.543
#25	Formamide	87300	154260	0‡	0.698
#26	Propylene Carbonate	584400	1358613	1	0.971
#27	γ-Butyrolactone (GBL)	239400	806003	1	0.688
#28	N-Methyl-2-Pyrrolidone (NMP)	1140	4019	0	1.016
#29	Thiazole	64800	373227	1	0.67
#30	N-Methyl Formamide	328200	1191267	1	0.44
#31	Diethylene Glycol	34830	7551	0	1.102
#32	Ethylenediamine	8820	49782	0	1.284
#33	Ethanolamine	674100	208622	1	0.985
#34	Benzyl Alcohol	1980	2193	0	1.452
#35	Ethylene Glycol	157980	56752	0	1.59
#36	Formic Acid	300	1150	0	1.211
#37	Acetic Acid	135	721	0	1.38
#38	Acetic Anhydride	251100	1764857	1	0.995
#39	Water (1% Soluble In - Ro=18.1)	184500	1244235	1‡	0.565
#40	1.3-Butanediol	68880	4246	0	1.492
#41	Dimethyl Formamide	55	416	0	0.772*
#42	Acetylacetone	690	5658	0	1.253
#43	Benzonitrile	3180	15386	0	1.263
#44	Methanol	22800	257391	1	1.347*
#45	2-Butanol	22800	45852	0	1.524
#46	Isobutyl Alcohol	340	622	0	1.572
#47	1-Hexanol	720	970	0	1.5
#48	Cyclohexanol	500400	53468	0	1.636
#49	Salicylaldehyde	300	702	0‡	1.099

† Threshold to categorize into good/poor solvents was set to *RST* = 200,000.

‡ The dispersion stability with water as well as discolored probe liquids was omitted from the dataset to calculate HSP via sphere fitting.

* Anomalies indicating poor solvents inside HSP sphere or good solvents outside HSP spehere.

Table S8 Sedimentation time for 0.8 g of Ta_2O_5-KCL powder in dispersions with 2 mL each of the probe liquids, relative sedimentation time, dispersion stability, and relative energy difference.

No.	Probe liquid	Sedimentation time, t_{sed} (sec)	Relative sedimentation time, *RST* (arb. Unit)	Dispersion stability (1: good, 0: poor)†	Relative energy difference, *RED*
#1	Dimethyl Carbonate	10	119	0	1.646
#2	Diethylene Glycol Monobutyl Ether	40	56	0	1.725
#3	Diethylene Glycol Monoethyl Ether	50	92	0	1.562
#4	Acetone	10	231	0	1.48
#5	Diethylene Glycol Monomethyl Ether	120	235	0	1.709
#6	Ethylene Glycol Monoethyl Ether	75	288	0	1.954
#7	Methyl Ethyl Ketone (MEK)	9	171	0	1.534
#8	Ethylene Glycol Monomethyl Ether	120	548	0	1.935
#9	n-Butyl Acetate	10	105	0	2.179
#10	1-Butanol	65	178	0	2.266
#11	Propylene Carbonate	160	436	0	1.005
#12	2-Propanol	40	143	0	2.322
#13	1,3-Dioxolane	10	118	0	1.36
#14	1-Nitropropane	70	614	0	1.093
#15	Glycerol	241200	1687	0	3.39
#16	Dimethyl Acetamide (DMA)	3360	25113	1	1.006*
#17	1,4-Dioxane	20	116	0	2.201
#18	Cyclohexanone	75	262	0	1.219
#19	Caprolactone (Epsilon)	55900	70057	1	0.517
#20	Dipropylene Glycol	25800	2278	0	2.01
#21	Ethanol	25	166	0	2.467
#22	Acetonitrile	570	12023	0	1.601
#23	Dimethyl Sulfoxide (DMSO)	47100	163230	1	0.642
#24	2-Chlorophenol	7920	15856	0	1.774
#25	Formamide	120	248	0	2.918
#26	N-Methyl Formamide	150	635	0	1.717
#27	γ-Butyrolactone (GBL)	32550	128241	1	0.663
#28	N-Methyl-2-Pyrrolidone (NMP)	7920	32591	1	0.574
#29	Dimethyl Formamide	120	1058	0	0.872*
#30	Methanol	5	66	0	2.94
#31	Ethylene Glycol	32550	13679	0	3.23
#32	1,3-Butanediol	7080	509	0	2.618
#33	Diethylene Glycol	730	185	0	2.131
#34	Cyclohexanol	91200	11359	0	2.054
#35	Benzyl Alcohol	120	155	0	1.67
#36	Acetic Anhydride	1260	10352	0	1.275
#37	Thiazole	6480	43758	1	1.029*
#38	Pyridine	1110	6352	0	0.980*
#39	Nitrobenzene	10	40	0	1.066
#40	Water (1% Soluble In - Ro=18.1)	10200	80248	1‡	2.339
#41	Methylene Diiodide (Diiodomethane)	80	141	0‡	2.013
#42	Acetic Acid	20	125	0	2.176
#43	Aniline	140	265	0	1.528
#44	Formic Acid	60	270	0	2.048
#45	Salicylaldehyde	2250	6166	0	0.907*
#46	Dichloroacetic Acid	48428.57143	61703	1	1.297*
#47	Acetol	120	190	0	1.567
#48	Ethanolamine	960	347	0	2.38
#49	Ethylenediamine	50	328	0	2.012
#50	Thiophenol	180	1090	0	1.691

† Threshold to categorize into good/poor solvents was set to *RST* = 16,000.
‡ The dispersion stability with water as well as discolored probe liquids was omitted from the dataset to calculate HSP via sphere fitting.
* Anomalies indicating poor solvents inside HSP sphere or good solvents outside HSP sphere.

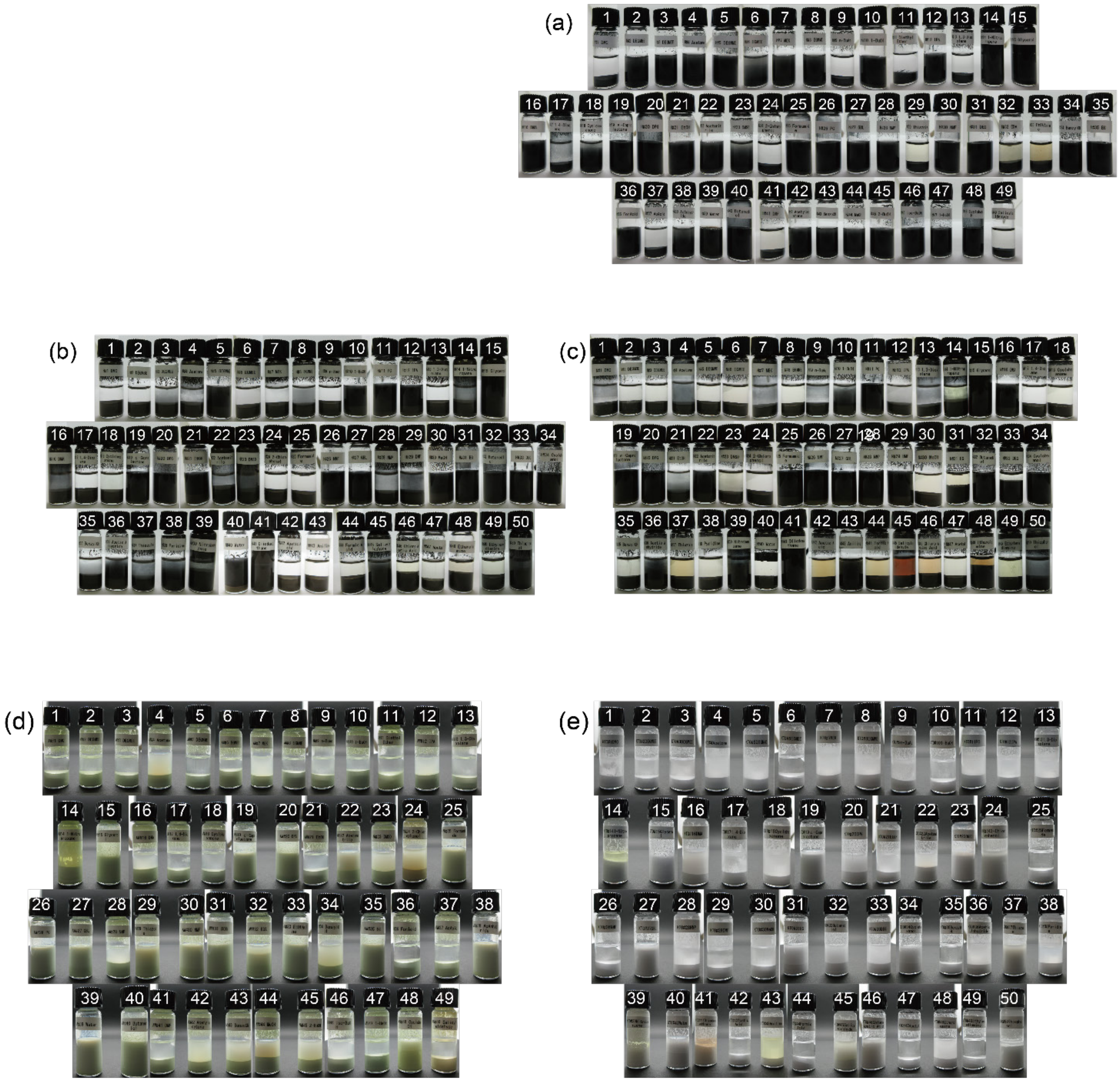

Fig. S3 Photographs of dispersions of (a) WC-HCS, (b) TaC-HCS, (c) TaC-JNM, (d) WO_3-AM, (e) Ta_2O_5-KCL powders in all the probe liquids after 10 min sonication and subsequent 1.5 h sedimentation.

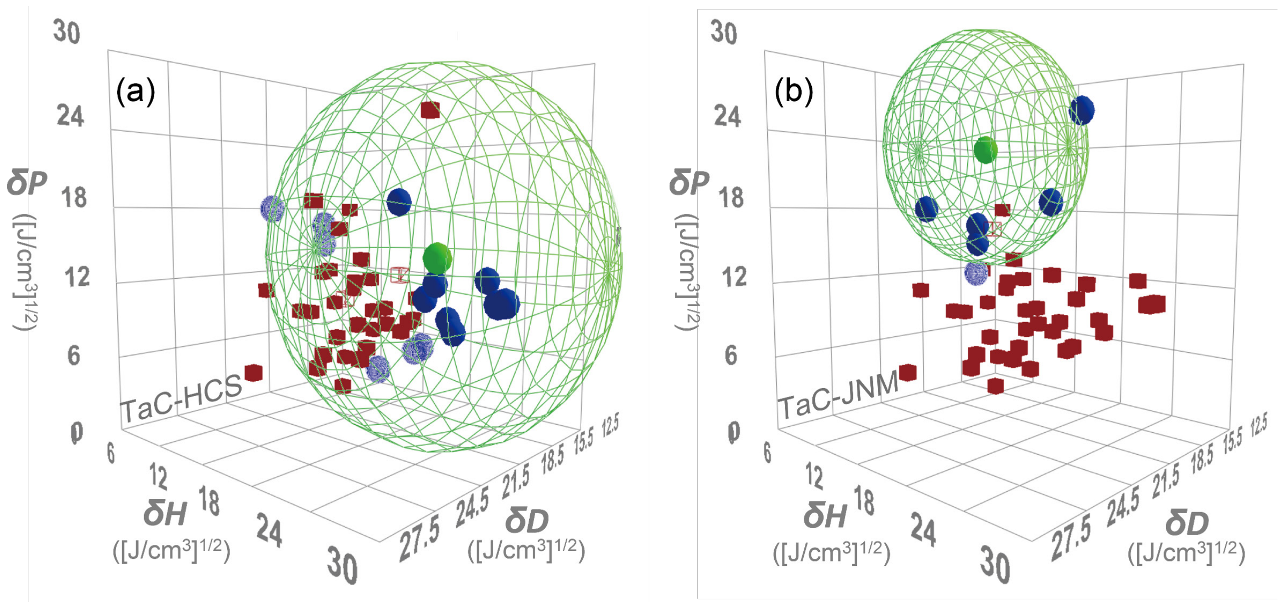

Fig. S4 Pseudo-3D plots of good (blue ●, ○) and poor (red ■, □) test solvents for (a) TaC-HCS and (b) TaC-JNM powders in HSP space (plots for the dispersions discolored during sedimentation test are excluded). The green wireframe sphere is the outer shell of HSP sphere fitted via sphere method, whose center (green ●) and radius correspond to HSP of TaC powder and its interaction radius, respectively. The solid and open (or shaded) plots stand for good/poor (normal) and poor/good (anomalous) solvents inside/outside the obtained HSP sphere, respectively.

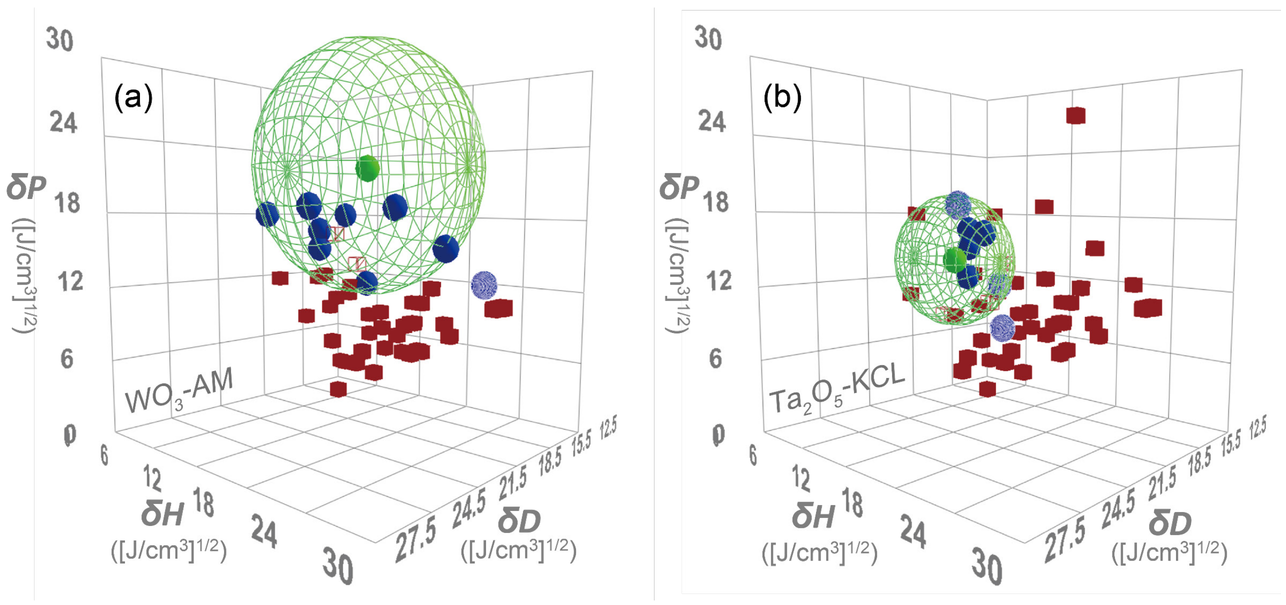

Fig. S5 Pseudo-3D plots of good (blue ●, ○) and poor (red ■, □) test solvents for (a) WO_3-AM and (b) Ta_2O_5-KCL powders in HSP space (plots for the dispersions discolored during sedim5entation test are excluded). The green wireframe sphere is the outer shell of HSP sphere fitted via sphere method, whose center (green ●) and radius correspond to HSP of WO_3-AM (or Ta_2O_5-KCL) powder and its interaction radius, respectively. The solid and open (or shaded) plots stand for good/poor (normal) and poor/good (anomalous) solvents inside/outside the obtained HSP sphere, respectively.

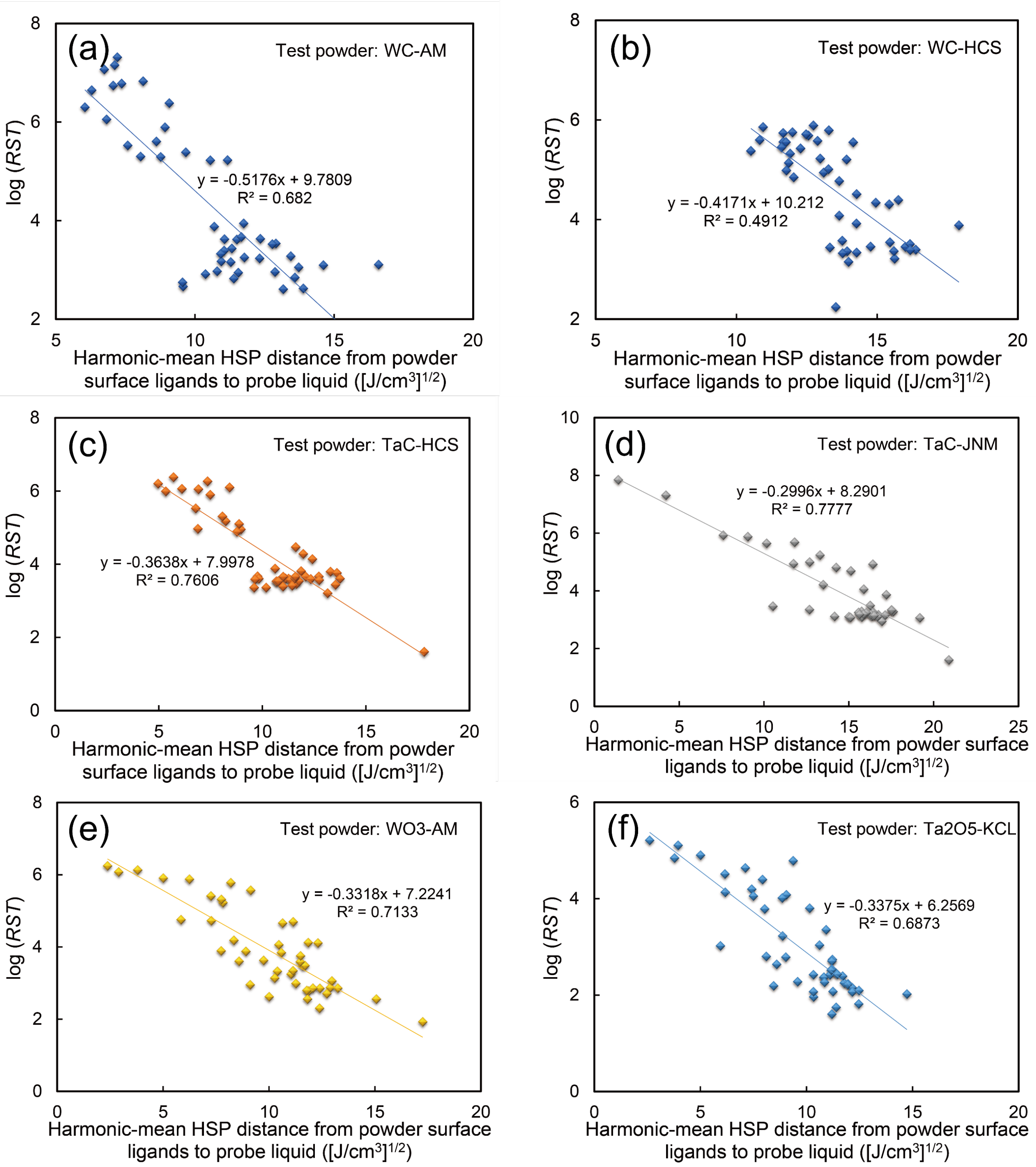

Fig. S6 Correlation of log (*RST*) to harmonic-mean HSP distance to probe liquid from 4–5 of surface ligands on the test powders of (a) WC-AM, (b) WC-HCS, (c) TaC-HCS, (d) TaC-JNM, (e) WO_3-AM, and (f) Ta_2O_5-KCL after regression with least-square method.

Table S9 Chemical bonds/surface ligands as well as their binding energy and atomic concentration identified in narrow-scan XPS spectra from test powders. The underlined compositions indicate possible top-most surface ligands (with high cohesive energy) governing dispersion stability.

Core level	Possible chemical bond/ligand	WC-AM		WC-HCS		TaC-HCS		TaC-JNM		WO_3-AM		Ta_2O_5-KCL	
		Binding energy (eV)	Compositision (at%)	Binding energy (eV)	Compositision (at%)	Binding energy (eV)	Compositision (at%)	Binding energy (eV)	Compositision (at%)	Binding energy (eV)	Compositision (at%)	Binding energy (eV)	Compositision (at%)
W4$f_{7/2}$	Total		29.0		30.7						25.4		
	W–C	31.7	14.2	31.7	15.6								
	W–N	32.5	3.3	32.5	3.6								
	W–O (WO_2)	33.2	3.0	33.2	2.4								
	W–O ($W_{18}O_{49}$)			34.8	0.9								
	W–O (WO_3)	35.8	6.7	35.8	7.0					35.8	24.8		
	Ammonium salt (e.g., APT: ammonium paratungstate)	36.9	**1.7**	36.9	**1.2**					36.9	**0.6**		
Ta4$f_{7/2}$	Total						27.2		25.5				25.7
	Ta–metal					21.9	2.1						
	Ta–N (Ta_2N)					22.4	2.0						
	Ta–N (TaN)					23.2	2.2						
	Ta–C					23.6	7.0	23.6	7.5				
	Ta–O (TaO_2/Ta_2O_3)					24.7	4.6	24.7	0.9				
	Ta–O (TaO_2)					25.5	2.2	25.3	4.7				
	Ta–O (Ta_2O_5)					26.3	7.1	26.4	8.2			25.8	25.7
	Ta–CO_3^{2-} (Ta[CO_3^{2-}]$_3$)							27.8	**4.2**				
N1s	Total		3.0		3.1						1.4		
	W–N	397.4	1.6	397.4	1.7								
	C–N=C	398.7	**0.2**	398.7	**0.3**								
	NH_2, N–C=O, C≡N	399.9*	**0.5**	399.9*	**0.4**					400.1**	**1.2**		
	NH_4^+	401.7	**0.6**	401.7	**0.6**								
	APT	402.3	**0.1**	402.3	**0.1**					402.3	**0.2**		
C1s	Total		36.4		35.0		33.6		31.7		9.7		10.7
	M–carbide (#1)	282.4	12.2	282.4	13.1	281.4	4.3						
	M–carbide (#2)	283.0	4.1	283.0	4.8	282.8	10.6	282.9	7.5				
	C (sp_2)	284.1	7.8	284.1	7.2	284.2	6.1						
	C (sp_3)	284.8	3.6	284.8	2.7	284.9	2.6	284.6	7.0	284.7	7.0	284.5	6.0
	C–N	285.3	**3.9**	285.3	**3.2**	285.6	**4.6**			285.3	**0.4**	285.1	**1.8**
	C–O–C, C–OH, C≡N	286.2†	**2.5**	286.2†	**1.9**	286.5†	**2.1**	286.1†	**8.2**	286.4††	**2.0**	286.2††	**1.9**
	C=O, N–C=O	287.7	**1.3**	287.7	**1.0**	288.0	**1.6**	287.5	**1.8**				
	N–C(=O)–N, C–O–C=O	288.6‡	**1.1**	288.6‡	**1.1**	288.8‡	**1.8**	288.6‡‡	**4.4**	288.5‡	**0.3**	288.3‡	**1.0**
	CO_3^{2-}							290.1	**2.6**				
O1s	Total		31.6		31.3		39.2		42.8		63.5		63.6
	M–oxide (#1)					528.9	8.3						
	M–oxide (#2)	530.8	19.4	530.7	16.6	530.2	18.5	530.3	16.4	530.4	51.0	529.9	57.8
	M–hydroxide, C=O	531.2	**8.0**	531.1	**10.7**	531.2	**9.2**	531.2	**1.0**	531.2	**12.5**	531.2	**5.8**
	CO_3^{2-}							531.6	**13.0**				
	H_2O, C–O–C, C–OH	532.5	**4.2**	532.4	**4.0**	532.5	**3.2**	532.5	**8.4**				
	O–C=O							533.7	**3.9**				

*assigned to NH_2 and/or N–C=O due to NH_3 as dominant desorbed gas in TDS analysis. **assigned to C≡N due to HCN as dominant desorbed gas in TDS analysis.

†assigned to be C–O–C and/or C–OH. ††assigned to be C≡N due to HCN as dominant desorbed gas in TDS analysis as well as the absence of C–O–C and/or C–OH in O1s.

‡assigned to N–C(=O)–N due to the absence of O–C=O in O1s. ‡‡assigned to C–O–C=O due to the presence of O–C=O in O1s.
